# Genetic variation at 16q24.2 is associated with small vessel stroke

**DOI:** 10.1002/ana.24840

**Published:** 2017-03-25

**Authors:** Matthew Traylor, Rainer Malik, Mike A. Nalls, Ioana Cotlarciuc, Farid Radmanesh, Gudmar Thorleifsson, Ken B. Hanscombe, Carl Langefeld, Danish Saleheen, Natalia S. Rost, Idil Yet, Tim D. Spector, Jordana T. Bell, Eilis Hannon, Jonathan Mill, Ganesh Chauhan, Stephanie Debette, Joshua C. Bis, W.T. Longstreth, M. Arfan Ikram, Lenore J. Launer, Sudha Seshadri, METASTROKE, UK Young Lacunar DNA Study, NINDS Stroke Genetics Network, Neurology Working Group of the CHARGE Consortium, Monica Anne Hamilton‐Bruce, Jordi Jimenez‐Conde, John W. Cole, Reinhold Schmidt, Agnieszka Słowik, Robin Lemmens, Arne Lindgren, Olle Melander, Raji P. Grewal, Ralph L. Sacco, Tatjana Rundek, Kathryn Rexrode, Donna K. Arnett, Julie A. Johnson, Oscar R. Benavente, Sylvia Wasssertheil‐Smoller, Jin‐Moo Lee, Sara L. Pulit, Quenna Wong, Stephen S. Rich, Paul I.W. de Bakker, Patrick F. McArdle, Daniel Woo, Christopher D. Anderson, Huichun Xu, Laura Heitsch, Myriam Fornage, Christina Jern, Kari Stefansson, Unnur Thorsteinsdottir, Solveig Gretarsdottir, Cathryn M. Lewis, Pankaj Sharma, Cathie L.M. Sudlow, Peter M. Rothwell, Giorgio B. Boncoraglio, Vincent Thijs, Chris Levi, James F. Meschia, Jonathan Rosand, Steven J. Kittner, Braxton D. Mitchell, Martin Dichgans, Bradford B. Worrall, Hugh S. Markus, on behalf of the International Stroke Genetics Consortium

**Affiliations:** ^1^ Department of Medical and Molecular GeneticsKing's College LondonLondonUnited Kingdom; ^2^ Institute for Stroke and Dementia Research, Klinikum der Universität MünchenLudwig‐Maximilians UniversityMunichGermany; ^3^ Laboratory of Neurogenetics, National Institute on AgingBethesdaMD; ^4^ Institute of Cardiovascular Research Royal Holloway University of London (ICR2UL)LondonUnited Kingdom; ^5^ Division of Neurocritical Care and Emergency Neurology, Massachusetts General HospitalBostonMA; ^6^ J. Philip Kistler Stroke Research Center, Department of NeurologyMassachusetts General HospitalBostonMA; ^7^ Center for Human Genetic Research, Massachusetts General HospitalBostonMA; ^8^ deCODE genetics/AMGENReykjavikIceland; ^9^ Center for Public Health Genomics and Department of Biostatistical Sciences, Wake Forest School of MedicineWinston‐SalemNC; ^10^ Department of Genetics, Perelman School of MedicineUniversity of PennsylvaniaPhiladelphiaPA; ^11^ Department of Twin Research & Genetic EpidemiologyKing's College LondonLondonUnited Kingdom; ^12^ University of Exeter Medical School, University of ExeterExeterUnited Kingdom; ^13^ Social, Genetic and Developmental Psychiatry Center, Institute of Psychiatry, Psychology and Neuroscience, King's College LondonLondonUnited Kingdom; ^14^ Inserm Research Center for Epidemiology and Biostatistics (U897)–Team NeuroepidemiologyBordeauxFrance; ^15^ University of BordeauxBordeauxFrance; ^16^ Cardiovascular Health Research Unit, Department of MedicineUniversity of WashingtonSeattleWA; ^17^ Departments of Neurology and EpidemiologyUniversity of WashingtonSeattleWA; ^18^ Department of Neurology, Epidemiology and RadiologyErasmus MC University Medical CenterRotterdamThe Netherlands; ^19^ Laboratory of Epidemiology and Population Sciences, National Institute on AgingBethesdaMD; ^20^ Boston University School of MedicineBostonMA; ^21^ Framingham Heart StudyFraminghamMA; ^22^ Department of NeurologyRoyal Adelaide HospitalAdelaideSouth AustraliaAustralia; ^23^ Neurovascular Research Group (NEUVAS), Neurology DepartmentInstitut Hospital del Mar d’Investigació MèdicaBarcelonaSpain; ^24^ Department of NeurologyUniversity of Maryland School of Medicine and Baltimore VAMCBaltimoreMD; ^25^ Department of Neurology, Clinical Division of NeurogeriatricsMedical University GrazGrazAustria; ^26^ Department of NeurologyJagiellonian UniversityKrakowPoland; ^27^ KU Leuven–University of Leuven, Department of NeurosciencesExperimental Neurology and Leuven Research Institute for Neuroscience and Disease (LIND)LeuvenBelgium; ^28^ VIB, Vesalius Research Center, Laboratory of Neurobiology, Department of NeurologyLeuvenBelgium; ^29^ University Hospitals LeuvenDepartment of NeurologyLeuvenBelgium; ^30^ Department of Clinical Sciences LundNeurology, Lund UniversityLundSweden; ^31^ Department of Neurology and Rehabilitation MedicineSkåne University HospitalLundSweden; ^32^ Department of Clinical SciencesLund UniversityMalmöSweden; ^33^ Neuroscience Institute, Saint Francis Medical Center, School of Health and Medical SciencesSeton Hall UniversitySouth OrangeNJ; ^34^ Department of Neurology, Miller School of MedicineUniversity of MiamiMiamiFL; ^35^ Harvard Medical School, Boston, MA, Center for Faculty Development and Diversity, Brigham and Women's HospitalBostonMA; ^36^ College of Public HealthUniversity of KentuckyLexingtonKY; ^37^ Department of Pharmacotherapy and Translational Research and Center for PharmacogenomicsUniversity of Florida, College of PharmacyGainesvilleFL; ^38^ Division of Cardiovascular Medicine, College of MedicineUniversity of FloridaGainesvilleFL; ^39^ Department of NeurologyUniversity of British ColumbiaVancouverBritish ColumbiaCanada; ^40^ Department of Epidemiology and Population HealthAlbert Einstein College of MedicineNew YorkNY; ^41^ Stroke Center, Department of NeurologyWashington University School of MedicineSeattleWA; ^42^ Department of Medical GeneticsUniversity Medical Center UtrechtUtrechtThe Netherlands; ^43^ Department of BiostatisticsUniversity of WashingtonSeattleWA; ^44^ Center for Public Health GenomicsUniversity of Virginia School of MedicineCharlottesvilleVA; ^45^ Julius Center for Health Sciences and Primary CareUniversity Medical Center UtrechtUtrechtThe Netherlands; ^46^ Department of Medical Genetics, Center for Molecular MedicineUniversity Medical Center UtrechtUtrechtThe Netherlands; ^47^ Department of MedicineUniversity of Maryland School of MedicineMD; ^48^ University of Cincinnati College of MedicineCincinnatiOH; ^49^ Program in Medical and Population Genetics, Broad InstituteBostonMA; ^50^ Division of Endocrinology, Diabetes and NutritionUniversity of Maryland School of MedicineBaltimoreMD; ^51^ Division of Emergency MedicineWashington University School of MedicineSt LouisMO; ^52^ The University of Texas Health Science Center at HoustonHoustonTX; ^53^ Institute of Biomedicinethe Sahlgrenska Academy at University of GothenburgGothenburgSweden; ^54^ Faculty of MedicineUniversity of IcelandReykjavikIceland; ^55^ Center for Clinical Brain Sciences & Institute of Genetics and Molecular MedicineUniversity of EdinburghEdinburghUnited Kingdom; ^56^ Nuffield Department of Clinical NeurosciencesUniversity of OxfordOxfordUnited Kingdom; ^57^ Department of Cerebrovascular DiseasesFondazione IRCCS Istituto Neurologico “Carlo Besta”MilanoItaly; ^58^ Department of NeurologyAustin Health and Florey Institute of Neuroscience and Mental HealthHeidelbergAustralia; ^59^ John Hunter Hospital, Hunter Medical Research Institute and University of NewcastleNewcastleNSWAustralia; ^60^ Department of NeurologyMayo Clinic JacksonvilleJacksonvilleFL; ^61^ Department of MedicineUniversity of Maryland School of MedicineBaltimoreMD; ^62^ Geriatrics Research and Education Clinical Center, Baltimore Veterans Administration Medical CenterBaltimoreMD; ^63^ Munich Cluster of Systems Neurology, SyNergyMunichGermany; ^64^ Departments of Neurology and Public Health SciencesUniversity of Virginia School of MedicineCharlottesvilleVA; ^65^ Stroke Research Group, Division of Clinical NeurosciencesUniversity of CambridgeCambridgeUnited Kingdom

## Abstract

**Objective:**

Genome‐wide association studies (GWAS) have been successful at identifying associations with stroke and stroke subtypes, but have not yet identified any associations solely with small vessel stroke (SVS). SVS comprises one quarter of all ischemic stroke and is a major manifestation of cerebral small vessel disease, the primary cause of vascular cognitive impairment. Studies across neurological traits have shown that younger‐onset cases have an increased genetic burden. We leveraged this increased genetic burden by performing an age‐at‐onset informed GWAS meta‐analysis, including a large younger‐onset SVS population, to identify novel associations with stroke.

**Methods:**

We used a three‐stage age‐at‐onset informed GWAS to identify novel genetic variants associated with stroke. On identifying a novel locus associated with SVS, we assessed its influence on other small vessel disease phenotypes, as well as on messenger RNA (mRNA) expression of nearby genes, and on DNA methylation of nearby CpG sites in whole blood and in the fetal brain.

**Results:**

We identified an association with SVS in 4,203 cases and 50,728 controls on chromosome 16q24.2 (odds ratio [OR; 95% confidence interval {CI}] = 1.16 [1.10–1.22]; *p* = 3.2 × 10^−9^). The lead single‐nucleotide polymorphism (rs12445022) was also associated with cerebral white matter hyperintensities (OR [95% CI] = 1.10 [1.05–1.16]; *p* = 5.3 × 10^−5^; N = 3,670), but not intracerebral hemorrhage (OR [95% CI] = 0.97 [0.84–1.12]; *p* = 0.71; 1,545 cases, 1,481 controls). rs12445022 is associated with mRNA expression of *ZCCHC14* in arterial tissues (*p* = 9.4 × 10^−7^) and DNA methylation at probe cg16596957 in whole blood (*p* = 5.3 × 10^−6^).

**Interpretation:**

16q24.2 is associated with SVS. Associations of the locus with expression of *ZCCHC14* and DNA methylation suggest the locus acts through changes to regulatory elements. Ann Neurol 2017;81:383–394

Genome‐wide association studies (GWAS) enable identification of common genetic variants that influence disease risk and have proved successful in elucidating pathophysiological mechanisms underlying diseases with a genetic influence.^1^ A number of GWAS associations have recently been identified with ischemic stroke, almost all of which have been associated with specific stroke subtypes.^2^, ^3^, ^4^ A number of genetic associations have been reported with cardioembolic (CE) and large artery stroke (LAS), but, in contrast, there have been no robust associations solely with small vessel stroke (SVS). This is despite epidemiological data that suggest genetic factors are particularity important for SVS. For example, there are a number of monogenic stroke disorders associated with SVS,^5^ and family history studies have shown a strong association between SVS and a family history of stroke.^6^ Similarly, related traits, including white matter hyperintensities (WMH), have been shown to have high heritability.^7^

SVS itself comprises one quarter of all ischaemic stroke and is one of the clinically overt manifestations of cerebral small vessel disease (SVD), the major cause of vascular cognitive impairment. Other radiological features of SVD include WMH, best observed on T_2_‐weighted magnetic resonance imaging (MRI), cerebral microbleeds—observed on gradient echo MRI, and intracerebral hemorrhages (ICH).^8^ Despite its importance, the pathogenesis of SVD remains poorly understood and this limits the development of proven treatments for established disease.

One consistent finding across adult‐onset neurological complex diseases, including Parkinson's disease,^9^ Alzheimer's disease,^10^ and stroke,^11^ is that younger onset cases have a stronger genetic burden from common disease‐associated single‐nucleotide polymorphisms (SNPs). Leveraging this increased burden, by focussing on younger‐onset cases in analysis of genetic data, can lead to detection of novel trait‐associated variants.^11^ This may be particularly relevant for SVS, given that epidemiological studies have shown stronger associations with SVS and a family history of stroke in younger stroke cases.^6^

Here, we perform an age‐at‐onset informed GWAS meta‐analysis in stroke, including a large population of younger‐onset (age < 70) SVS cases. We perform analysis for all ischemic stroke (IS) and its three subtypes: CE, LAS, and SVS. Using this approach, we identify a novel association with SVS, seek further validation of the locus in other SVD phenotypes, and assess the influence of SNPs at the locus on messenger RNA (mRNA) expression of nearby genes and DNA methylation at nearby CpG sites.

## Materials and Methods

### Study Design

**Figure 1:**
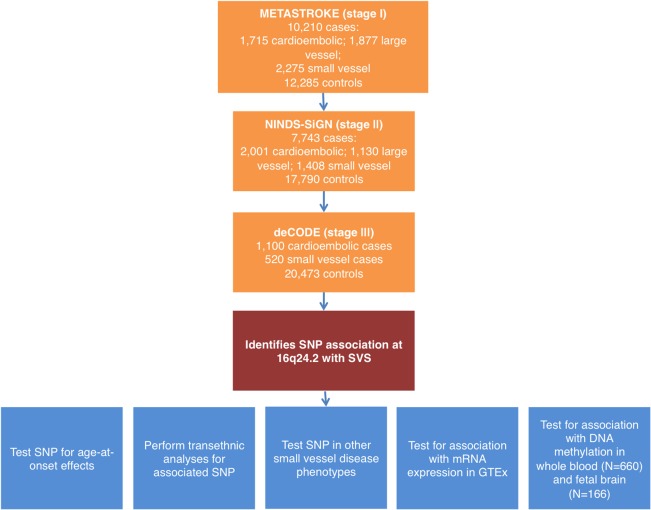
Flow chart of analyses performed. GTEx = Genotype‐Tissue Expression; mRNA = messenger RNA; SNP = single‐nucleotide polymorphism; SVS = small vessel stroke; [Color figure can be viewed at wileyonlinelibrary.com]

We used a three‐stage design for the association analysis (Fig 1). In brief, in stage I, we performed association analysis of stroke phenotypes in 10,210 cases and 12,285 controls of European ancestry from Europe, United States, and Australia; most of which contributed to the METASTROKE ischaemic stoke GWAS meta‐analysis—and all of which have been described previously (Table 1).^2^, ^12^, ^13^ In all cases, diagnosis of stroke was based on clinical evaluation with radiological confirmation. Subtyping of stroke cases was based on the TOAST criteria; in this analysis, we considered the CE, LAS, and SVS subtypes.^14^ Of note, our SVS analysis included a large sample (1,012 cases, 970 controls) of younger‐onset (age < 70) MRI‐confirmed lacunar strokes, meaning that, although we investigated all subtypes, we had most power to identify associations with SVS.

**Table 1:** Ischemic Stroke Study Participants

Population	IS	CE	LAS	SVS	Controls	% Cases With MRI	Age of Cases (mean (SD))
Stage I populations							
ASGC	1,162	240	421	310	1,244	43.0	72.9 (13.2)
WTCCC2‐Germany	1,174	330	346	106	797	83.0	66.7 (12.9)
WTCCC2‐UK	2,374	474	498	460	5,175	37.2	72.2 (12.5)
Milano	366	64	73	25	407	86.7	57.4 (15.6)
DNA‐lacunar/GENESIS	1,287	80	64	1,012	970	100.0	59.6 (12.0)
LSS	455	157	70	55	455	89.0	67.7 (14.5)
ISGS/SWISS	1,014	235	217	187	1,370	83.0	66.5 (13.6)
BRAINS	361	29	120	97	444	30.8	74.4 (14.2)
MGH‐GASROS	294	106	68	23	376	60.0	66.7 (14.5)
VISP	1,723	—	—	—	1,047	47.0	68.0 (10.7)
Total (discovery)	10,210	1,715	1,877	2,275	12,285		
Stage II populations							
NINDS Stroke Genetics Network	7,743	2,001	1,130	1,408	17,970	62.0	66.3 (14.8)
Stage III populations							
deCODE	—	1,100	—	520	20,473	NA	72.7 (11.6)
Total	17,953	4,816	3,007	4,203	50,728		

IS = all ischemic stroke; CE = cardioembolic stroke; LAS = large artery stroke; SVS = small vessel stroke; ASGC = Australian Stroke Genetics Collaborative; WTCCC2 = Wellcome Trust Case Control Consortium 2; LSS = Leuven Stroke Study; BRAINS = Bio‐repository of DNA in stroke; MGH‐GASROS = The MGH Genes Affecting Stroke Risk and Outcome Study; VISP = The Vitamin Intervention for Stroke Prevention Trial; NA = information not available.

In stage II, we took three SNPs from the top 25 loci from each phenotype forward for a first in silico replication in the NINDS Stroke Genetics Network (SiGN),^2^ which consisted of 7,743 cases and 17,790 controls. We meta‐analyzed stages I and II together and identified three loci with *p* < 5 × 10^−7^. Finally, in stage III, we determined whether these three SNPs were associated with the phenotype in which they were identified (CE or SVS) by in silico replication in a large Icelandic population (deCODE; 520 SVS cases, 1,100 CE cases, 50,728 controls; stage III).

### Genotyping and Imputation

Genotyping, quality control, and imputation of all studies has been described previously.^2^, ^3^, ^13^ All studies were genotyped on commercially available arrays from Illumina (San Diego, CA) or Affymetrix (Santa Clara, CA) and imputed to 1000 Genomes phase 1 reference panels using IMPUTE or MACH.^15^ Imputation quality score was assessed by calculating the ratio of the observed to the expected binomial variance of the allele dosage.

### Association Analysis

Association analysis was performed using a covariate‐informed approach,^11^, ^16^ which we, and others, have implemented previously.^11^, ^17^ Briefly, the approach uses case/control status and a covariable—in this case, age‐at‐onset—to estimate each individual's stroke liability, which can be interpreted as their underlying propensity to stroke, on a normally distributed scale. In this analysis, cases with an earlier age‐at‐onset take more‐extreme positive values than late‐onset cases given that, attributed to the lower prevalence of stroke at younger ages, they are assumed to have higher stroke liability. Conversely, controls who are older and stroke free at age‐at‐observation take more‐extreme negative value than younger controls given that they have been stroke free for a longer time and are therefore assumed to have a lower stroke liability.

In this analysis, the approach was implemented in our software, CIAO (provided at https://sites.google.com/site/mtraylor263/software/covariate-informed-gwas-analysis). Specifically, the approach taken is to model phenotype data using a continuous unobserved normally distributed quantitative trait, called the disease liability ( $\varphi =\sum_{j=1}^{J}c_{j}\left(t_{j}-\bar{t_{j}}\right)+m+\varepsilon$), where ε=γg+N(0,1) and g denotes the genetic effects. Then, an individual is a case (*z = 1*) if and only if φ≥0 and is a control (*z = 0*) otherwise. $c_{j}$ is a parameter estimating the effect of a given covariate *j* on the liability scale. m denotes the disease prevalence *p* at the covariate mean $\bar{t_{j}}$ under a normal cumulative distribution function (Φ(−m)=p). This model is used to approximate the effect of a disease covariate—in this case, age‐at‐onset—on the liability scale, based on estimates of risk of IS by age from epidemiological data, thereby estimating $c_{j}$. For this analysis, the sex‐specific risk of IS by age from an index age of 55 was obtained from population‐based estimates (1.8%, 5.4%, and 12.1% before 65, 75, and 85, respectively, in women; 2.4%, 7.3%, and 12.6% before 65, 75, and 85, respectively, in men).^18^ We assumed that 20% of IS cases had each of the cardioembolic, small vessel, or large vessel stroke subtypes, approximating proportions observed in population‐based studies.^19^ We developed two models for our analysis; one based on the risk rates for all IS and, second, for the three stroke subtypes. We used these models to calculate posterior mean liabilities after conditioning on age‐at‐onset for the four stroke phenotypes separately ( $E\left(\varepsilon |z,t\right)=\frac{\int_{-c\left(t-\bar{t}\right)-m}^{\infty }\varepsilon \frac{1}{\sqrt{2\pi }}e^{\left(\frac{-\varepsilon^{2}}{2}\right)}d\varepsilon }{\int_{-c\left(t-\bar{t}\right)-m}^{\infty }\frac{1}{\sqrt{2\pi }}e^{\left(\frac{-\varepsilon^{2}}{2}\right)}d\varepsilon },\;if\;z=1$). Controls were modeled in the same way, but were assumed to take the posterior mean from the lower (unaffected) portion of the distribution in the liability threshold model ( $E\left(\varepsilon |z,t\right)=\frac{\int_{\infty }^{-c\left(t-\bar{t}\right)-m}\varepsilon \frac{1}{\sqrt{2\pi }}e^{\left(\frac{-\varepsilon^{2}}{2}\right)}d\varepsilon }{\int_{\infty }^{-c\left(t-\bar{t}\right)-m}\frac{1}{\sqrt{2\pi }}e^{\left(\frac{-\varepsilon^{2}}{2}\right)}d\varepsilon },\;if\;z=0)$. Where age data was missing, individuals were assigned the median age value (<1% of cases). Regression was then performed on posterior liabilities ( E(ε|z,t)) by multiplying the number of samples by the squared correlation between the expected genotype dosage and posterior mean liabilities for each of the discovery cohorts in the four IS phenotypes (all IS, CE, LAS, and SVS). Ancestry‐informative principal components were included, where appropriate, using the EIGENSTRAT procedure.^20^ Any residual inflation was accounted for by adjusting results by the genomic inflation factor, λ.^21^ In all analyses, SNPs with imputation quality score <0.7 or minor allele frequency (MAF) < 0.01 were excluded and meta‐analysis was performed using Stouffer's method in METAL.^22^

### Further Analysis of a Novel Locus Associated With SVS

For a novel variant associated with SVS, we performed further analysis to elucidate the association for different groups based on age‐at‐onset. First, for data sets in stage I and II, we divided the cases into quartiles based on age‐at‐onset and estimated the association of the SNP with each quartile using logistic regression with all controls, meta‐analyzing using a fixed‐effects inverse variance weighted approach (data not available in BRAINS, MGH‐GASROS, and ISGS/SWISS). Second, we interrogated associations at the locus in non‐European ancestry populations, comprising 657 small vessel African‐American stroke cases and 3,251 matched controls from the NINDS Stroke Genetics Network and African or African‐Caribbean ancestry individuals from the South London Ethnicity and Stroke Study (SLESS),^2^, ^23^ and 314 SVS cases and 5,193 controls of Pakistani ancestry from the RACE study.^3^ We used logistic regression to evaluate the association within each group and evaluated the overall transethnic association by meta‐analyzing using Stouffer's method.

In addition, we explored the association of the SNP with other SVD phenotypes. We evaluated association of the SNP with (1) white matter hyperintensity volumes (WMHV) measured on T_2_‐weighted MRI in 3,670 IS patients of European ancestry^24^; (2) in MRI‐defined small subcortical brain infarcts (SSBI) brain infarcts in 17,197 transethnic individuals (85.7% European; 8.8% African‐American; 3.5% Hispanic; 1.0% Chinese; and 1.0% Malay) from community studies recruited within the neuro‐CHARGE consortium (mean age, 68.90 ± 10.31; 1,986 with infarcts). SSBI were defined as MRI‐defined brain infarcts of 3 to 15 or 3 to 20mm in size, located in the basal ganglia, the white matter, or the brainstem. Association analysis was performed overall, and for the subset of cases with extensive WMH burden—defined as the top age‐specific quartile of WMHV on a quantitative scale or above the age‐specific median by 5‐year age categories for studies using semiquantitative measurements of WMH burden; N = 549; and (3) ICH in 1,545 European ancestry cases and 1,481 controls, described previously,^25^ and stratified according to lobar or nonlobar location.

### Evaluation of Regulatory Chromatin States, mRNA Expression, and DNA Methylation

To investigate a novel locus, we used existing resources and performed some further analyses to characterize its regulatory potential. We interrogated chromatin states and regulatory motifs from ENCODE and Epigenomics Roadmap using Haploreg v4.1.^26^ We also evaluated whether the associated SNP influences gene expression using the Genotype‐Tissue Expression (GTEx) portal.^27^ Upon identifying an association between the SNP and expression of a nearby gene, we evaluated the evidence that the association signal for SVS and gene expression derives from the same causal variant using a Bayesian colocalization test.^28^ Using the R coloc package (http://cran.r-project.org/web/packages/coloc), we compared five models for SNPs with 50kb of our lead SNP using the approach (H_0_: No association with either trait; H_1_: Association with SVS, not with expression; H_2_: Association with expression, not with SVS; H_3_: Association with SVS and expression, two independent SNPs; H_4_: Association with SVS and expression, one shared SNP).

Next, we assessed whether the lead SNP (rs12445022), or three SNPs in linkage disequilibrium (LD; rs4843625, rs12920915, and rs12444224), influence DNA methylation levels in whole blood. We evaluated genetic associations of whole‐blood DNA methylation levels at selected CpG sites profiled on the Illumina Infinium HumanMethylation450 BeadChip array in a group of 660 monozygotic (MZ) female twins (mean age, 59; age range, 18–79). These individuals were research volunteers from the TwinsUK cohort in the United Kingdom.^29^ All were of European ancestry. For each CpG site of interest, we calculated the normalized methylation means for the 330 MZ twin pairs as a phenotype in the genetic analysis and took into account covariates, including smoking, body mas index, age, methylation plate, and blood‐cell count estimates. TwinsUK imputed genotypes were obtained for the 1000 Genomes reference set,^30^ where we excluded SNPs with Hardy–Weinberg *p* < 1 × 10^−4^, MAF < 5%, and those with IMPUTE info value <0.8. We tested for association with our SNP, or SNPs in close LD (r^2^ > 0.6) with DNA methylation at CpG sites. We used *p* < 4 × 10^−5^, equivalent to a false discovery rate (FDR) < 5%,^31^ to identify significant cis‐mQTL associations.

Finally, we explored genetic associations at 16q24.2 (defined as within 50kb of rs12445022) with DNA methylation profiles in 166 human fetal brain samples (92 male, 74 female) ranging from 56 to 166 days postconception initially using publically available data—which hold results for mQTL associations reaching the study‐wide significance threshold (http://epigenetics.essex.ac.uk/mQTL/). Methods for this study have been published in detail elsewhere.^32^ Briefly, DNA methylation levels were profiled on the Illumina Infinium HumanMethylation450 BeadChip array and SNP genotypes were obtained from the Illumina HumanOmniExpress BeadChip and imputed to 1000 Genomes phase 3 using SHAPEIT and Minimac3 through the Michigan Imputation Server.^15^, ^33^ SNP‐methylation probe pairs were tested using the R package, MatrixEQTL,^34^ including covariates to control for age, sex, and ancestry‐informative principal components. Upon identifying a significant association at 16q24.2, we performed additional analyses (not publicly available: we gained access to the data) to test whether any of our four SNPs (rs12445022, rs4843625, rs12920915, and rs12444224) were associated with methylation at the identified probe. We again used *p* < 4 × 10^−5^, equivalent to an FDR <5%,^31^ to identify significant cis‐mQTL associations.

## Results

### Association Analysis

In phase I association analysis, we confirmed previous associations between *HDAC9* and LAS (rs2107595, *p* = 3.0 × 10^−8^) and between *PITX2* and CE (rs192172299, *p* = 2.0 × 10^−9^).^3^, ^4^ Previous associations between *ZFHX3* and CE and between *MMP12* and LAS did not reach genome‐wide significance in this analysis (rs879324, *p* = 5.0 × 10^−7^ and rs586701, *p* = 0.0014, respectively).^11^ An SNP in a region close to *HABP2* previously associated with young‐onset IS was also significant, albeit not genome wide, in this analysis (rs11196288; *p* = 2.4 × 10^−4^).^35^ Genomic inflation λ and the equivalent values, scaled to 1,000 cases and 1,000 controls (λ_1000_),^36^ were well controlled across all analyses (IS, λ (λ_1000_) = 1.05 (1.00); CE, λ (λ_1000_) = 1.02 (1.00); LAS, λ (λ_1000_) = 1.02 (1.00); SVS. λ (λ_1000_) = 1.01 (1.00)).

**Figure 2:**
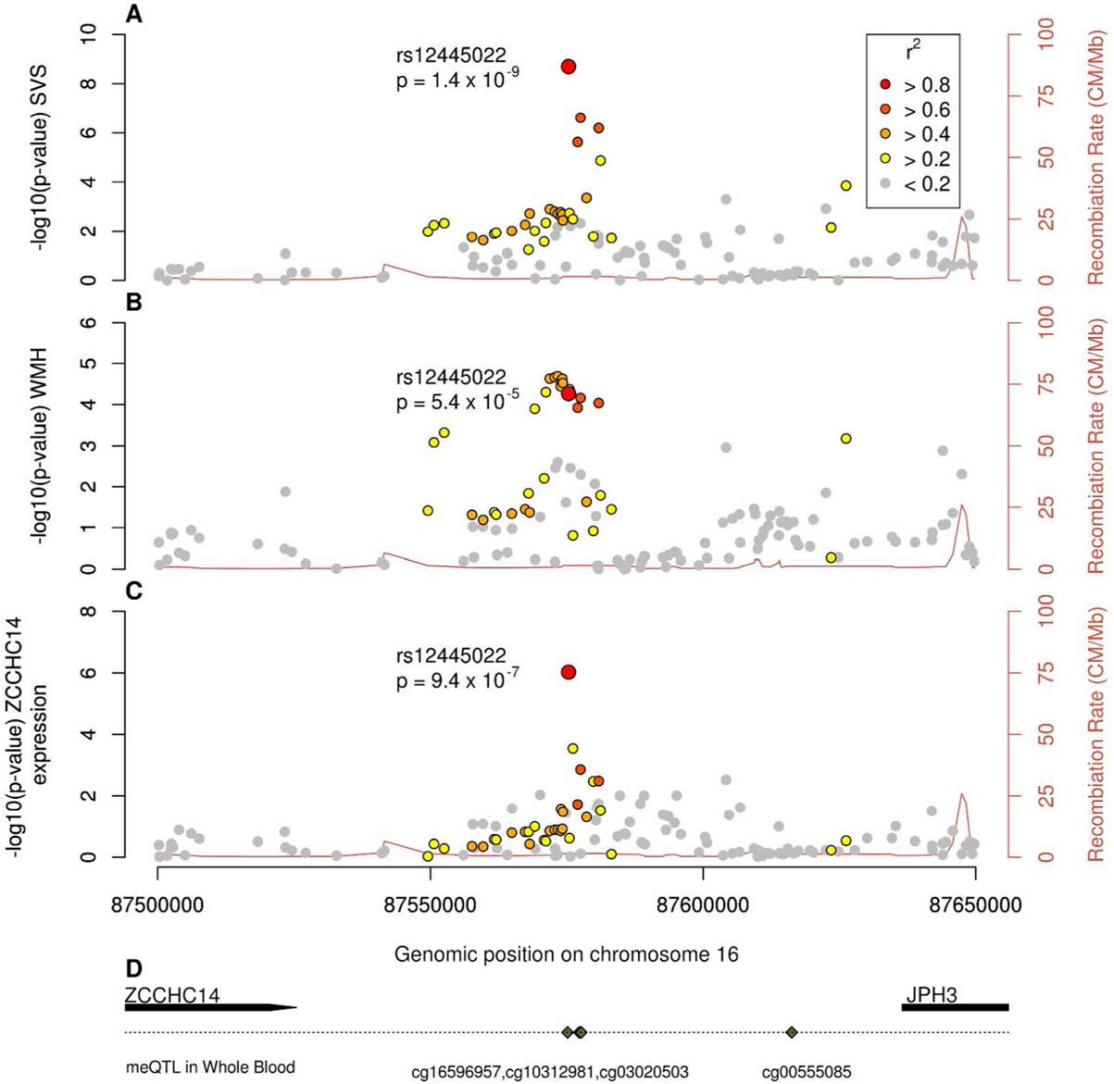
Associations at 16q24.2 with (A) small vessel stroke, (B) cerebral white matter hyperintensities, (C) mRNA expression of ZCCHC14, and (D) gene locations and associations of the locus with DNA methylation. mRNA = messenger RNA; SVS = small vessel stroke; WMH = white matter hyperintensities; ZCCHC14 = zinc finger CCHC domain‐containing 14; JPH3 = junctophilin 3; meQTL = methylation quantitative trait locus. [Color figure can be viewed at wileyonlinelibrary.com]

**Figure 3:**
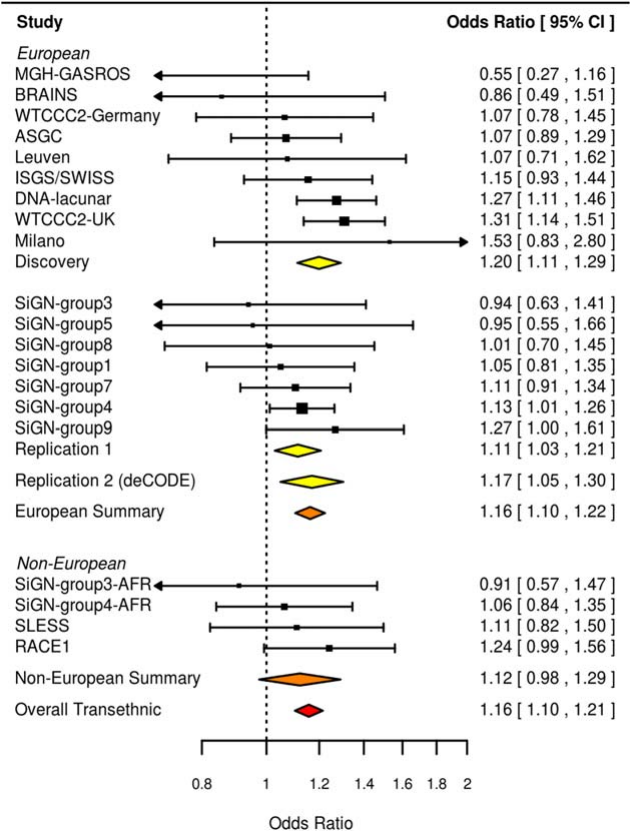
Forest plot of associations with rs12445022 under a logistic regression model. CI = confidence interval. [Color figure can be viewed at wileyonlinelibrary.com]

We took 25 independent loci forward (three SNPs in LD from each locus selected on *p* value) from each analysis (IS, CE, LAS, and SVS) for in silico replication in the NINDS Stroke Genetics Network study (stage II). Information on these SNPs is provided in Supplementary Tables 1 to 4. Following this analysis, excluding previously reported associations, three loci showed significance at *p* < 5 × 10^−7^ (two with SVS, one with CE) and one was genome‐wide significant (rs12445022, *p* = 4.4 × 10^−8^, associated with SVS). We followed up all three loci in a second in silico replication (stage III) in a large Icelandic population (deCODE). A single SNP, rs12445022, showed evidence of replication (*p* = 0.011). When performing a meta‐analysis across all populations, rs12445022 was associated with SVS at genome‐wide significance (*p* = 3.2 × 10^−9^; Fig 2). The SNP was either genotyped or well imputed (info > 0.9) in all cohorts and lies in an intergenic region between junctophilin 3 (*JPH3*) and zinc finger CCHC domain‐containing 14 (*ZCCHC14*). To confirm the association with rs12445022, we repeated the analysis using logistic regression, the approach taken in a conventional GWAS. The association was validated using this method, and associations were consistent across populations (odds ratio [OR; 95% confidence interval {CI}] = 1.16 [1.10–1.22]; *p* = 1.3 × 10^−8^; heterogeneity, *p* = 0.56; Fig 3).

### Further Analysis of a 16q24.2 Novel Locus Associated With SVS

**Figure 4:**
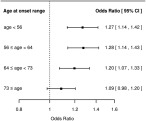
Association of rs12445022 with small vessel stroke by quartiles of age‐at‐stroke onset in Europeans. CI = confidence interval.

We evaluated association of the lead SNP in different quantiles of age at stroke onset, using all controls in each analysis. The strongest associations were observed in younger‐onset cases, suggesting that the influence of the SNP might be greatest in these individuals (Fig 4). However, this was not demonstrated statistically (*p* > 0.05).

**Figure 5:**
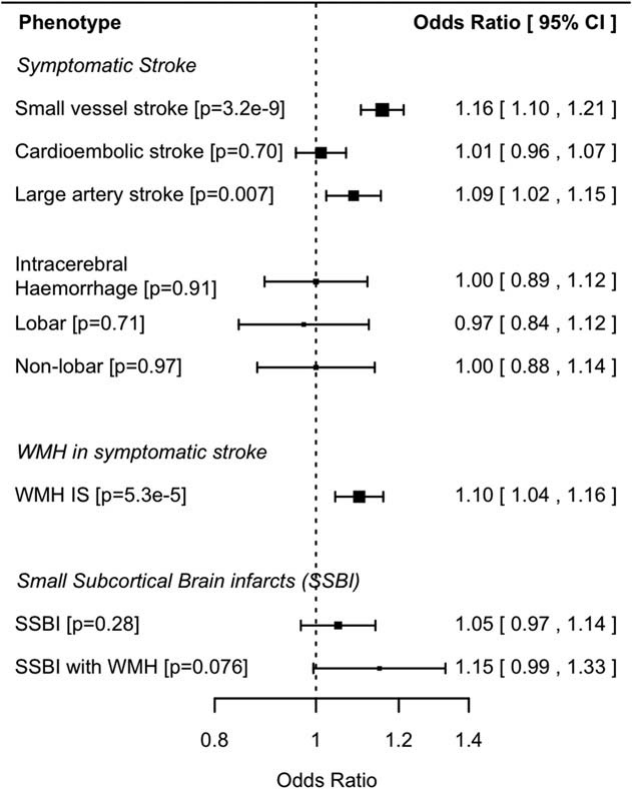
Associations with rs12445022 for stroke and cerebral small vessel disease phenotypes. CI = confidence interval; IS = all ischemic stroke; WMH = white matter hyperintensities.

We performed further analysis to assess whether the SVS‐associated SNP influenced other manifestations of cerebral SVD. The SNP (rs12445022) was also associated with increased T_2_‐WMHV (OR [95% CI] = 1.10 [1.05–1.16]; *p* = 5.3 × 10^−5^; Figs 2 and 5) and showed little heterogeneity across study groups (heterogeneity, *p* = 0.58). Conversely, the SNP was not associated with ICH—neither overall, nor in subgroups divided by lobar/nonlobar location. For SSBI, the direction of effect was the same as for SVS, but the effect was weaker and nonsignificant (OR [95% CI] = 1.05 [0.97–1.14]; *p* = 0.28). For the subgroup with WMH, the effect was stronger—and similar to that observed for SVS, but was again nonsignificant (OR [95% CI] = 1.15 [0.99–1.33]; *p* = 0.076).

We next evaluated the identified locus in non‐European ancestry populations. The SNP had a similar frequency to Europeans in South Asians from RACE (MAF = 37%), but was rarer in African ancestry populations, consisting of African Americans from the NINDS Stroke Genetics Network and United Kingdom individuals of African or African‐Caribbean ethnicity from SLESS (MAF = 14%). Associations with the SNP were in the same direction as in European ancestry populations (Fig 4), but did not reach statistical significance in either ancestry, reflecting the much smaller sample sizes. However, when combining data from all populations, evidence for association at the SNP (*p* = 1.4 × 10^−9^) was stronger than in European ancestry populations alone, which might suggest a common association across populations. Indeed, there was no evidence of a significant difference in the strength of association between the European and non‐European ancestry individuals (*p* = 0.64).

### Regulatory Chromatin States, mRNA Expression, and DNA Methylation Related to 16q24.2

We used existing databases to assess the functional consequences of SNPs in the 16q24.2 region. First, we used the Haploreg v4.1 database to interrogate chromatin states and regulatory motifs from ENCODE and the NIH Roadmap Epigenomics Mapping Consortium.^26^, ^37^, ^38^ The database showed that our lead SNP influences chromatin states in multiple tissues. The SNP is classified as a genic promoter in nine tissues, an enhancer in 13 tissues, and overlaps DNAse1 hypersensitivity sites in 21 tissues.

Second, we used publicly available databases to evaluate the evidence that the lead SNP influences expression of nearby genes using the GTEx portal.^27^ The implicated A allele of our lead SNP (rs12445022) was associated with decreased expression of *ZCCHC14* in tibial arterial tissue (*p* = 9.4 × 10^−7^; Fig 2). We used a Bayesian colocalization technique to assess whether the same variant drives the both the SVS association signal and mRNA expression of *ZCCHC14*.^28^ There was overwhelming evidence in support of H_4_ (posterior probability = 99.7%), strongly indicating that a single variant—most likely to be rs12445022—influences both SVS and expression of *ZCCHC14*.

Finally, we performed analyses to assess whether the lead SNP, or the three SNPs in LD, influence DNA methylation at CpG probes in whole blood. We found evidence that the lead SNP, and three SNPs in close LD (r^2^ > 0.6), influence DNA methylation at 4 nearby CpG sites (cg16596957, cg10312981, cg03020503, and cg00555085; all *p* < 4.0 × 10^−5^; Table 2). The implicated A allele of rs12445022 was associated with decreased methylation at the cg16596957 probe (beta [standard error; SE] = –0.38 [0.082]; *p* = 5.3 × 10^−6^). The SNPs explained between 5% and 8% of the methylation variance at the given CpG sites. The same 16q24.2 region by CpG probe (cg16596957) association was also recently reported in another study in whole blood.^40^ In addition, we looked for an association between SNPs at the 16q24.2 locus and DNA methylation levels in fetal brains, initially using publicly available data (http://epigenetics.essex.ac.uk/mQTL/). There was a strong association with SNPs in distant LD with our lead SNP (rs8047314 ∼ cg08031982; *p* = 7.1 × 10^−14^; r^2^ = 0.16 with rs12445022). We then performed additional analyses (not publicly available: we gained access to the data) to test whether our lead SNP, or the SNPs in close LD, were associated with methylation at cg08031982. We could identify no associations that reached our significance threshold (*p* < 4.0 × 10^−5^). However, there was a near‐significant association of rs12920915 and rs4843625 with methylation at the cg08031982 probe (both *p* = 7.8 × 10^−5^). Our lead SNP, rs12445022, was not associated (*p* = 9.9 × 10^−4^).

**Table 2:** Significant Associations Between rs12445022 and LD SNPs (r^2^ > 0.6) With cis‐Methylation Probes in Whole Blood

SNP Variant	SNP BP	CpG Probe	Probe BP	RA	Beta (SE)	r^2^	*p*
rs12445022	87,575,332	cg16596957	87,575,151	A	–0.38 (0.082)	0.058	5.3 × 10^−6^
rs4843625	87,576,996	cg16596957	87,575,151	C	–0.33 (0.075)	0.053	1.3 × 10^−5^
rs4843625	87,576,996	cg10312981	87,577,304	C	0.39 (0.074)	0.077	1.9 × 10^−7^
rs4843625	87,576,996	cg03020503	87,577,656	C	0.35 (0.075)	0.059	5.0 × 10^−6^
rs4843625	87,576,996	cg00555085	87,616,248	C	0.34 (0.075)	0.057	6.6 × 10^−6^
rs12920915	87,577,521	cg16596957	87,575,151	T	‐0.38 (0.075)	0.069	7.3 × 10^−7^
rs12920915	87,577,521	cg10312981	87,577,304	T	0.38 (0.075)	0.068	1.0 × 10^−6^
rs12920915	87,577,521	cg03020503	87,577,656	T	0.34 (0.076)	0.055	1.1 × 10^−5^
rs12920915	87,577,521	cg00555085	87,616,248	T	0.33 (0.076)	0.051	2.2 × 10^−5^
rs12444224	87,580,855	cg16596957	87,575,151	T	–0.38 (0.075)	0.068	8.0 × 10^−7^
rs12444224	87,580,855	cg10312981	87,577,304	T	0.38 (0.075)	0.069	8.7 × 10^−7^
rs12444224	87,580,855	cg03020503	87,577,656	T	0.35 (0.076)	0.054	1.1 × 10^−5^
rs12444224	87,580,855	cg00555085	87,616,248	T	0.32 (0.076)	0.050	2.6 × 10^−5^

SNP = single‐nucleotide polymorphism; BP = base position; RA = reference allele; SE = standard error; r^2^ = proportion of methylation variance explained by respective genotype.

## Discussion

Genome‐wide association studies in SVS have largely been disappointing. Some studies have suggested that an association with all IS at the highly pleiotropic 12q24.12 is driven by an association with SVS,^2^ but no genome‐wide significant associations specifically with SVS have yet been identified. Using an age‐of–onset informed analysis approach, we identified a novel locus at 16q24.2 associated with SVS. The SNP was also associated using a standard logistic regression approach, but was less significant—a difference of almost an order of magnitude (*p* = 3.2 × 10^−9^ compared to *p* = 1.3 × 10^−8^). In addition, the association was stronger with younger‐onset SVS, suggesting a greater influence in these individuals. We tested whether the 16q24.2 association extends to other cerebral SVD‐related phenotypes. We showed that the same locus also influences WMH and may have a similar effect on MRI‐defined subcortical brain infarcts from prospective studies, although the association did not reach significance in our analysis. However, the locus does not appear to influence risk of ICH. A SNP in the same 16q24.2 region (rs4081947), in partial LD with our SNP (r^2^ = 0.28), was also recently reported to be associated with migraine in a large GWAS meta‐analysis.^41^ These data provide strong supportive evidence that this 16q24.2 locus harbors variants that influence diseases of the cerebral vasculature.

Identifying the mechanisms by which GWAS associations influence disease risk presents additional challenges. In this case, the underlying mechanism and the specific genes implicated remains uncertain. Interrogation of mRNA expression data points to the lead SNP influencing expression of the nearest gene, *ZCCHC14*. This gene is ubiquitously expressed, but is highly expressed in arterial tissues and in the brain. However, its function is not well characterized. Zinc fingers of the CCHC‐type contain an 18‐digit residue found in the nucleocapsid of retroviruses and therefore may be important in viral response. Other plausible candidate genes reside nearby. The locus lies around 1Mb away from genes encoding forkhead box proteins, including *FOXC2, FOXL1*, and *FOXF1*. These proteins, particularly the closely related *FOXC1*—a paralogue of *FOXC2*, have been implicated in Mendelian forms of SVS.^42^ We found no evidence linking our SNP to expression of these genes. However, the function of these proteins changes dramatically between early development and in adult tissues,^43^ which might explain the absence of an association. This, coupled with the fact that *FOXF2* variants have also recently been implicated in IS,^44^ make forkhead box proteins exciting targets for follow‐up experiments.

Assessing DNA methylation, the process by which methyl groups are added to DNA thereby modifying its function, offers another potential method for mechanisticinsight. This epigenetic process influences gene expression and regulation in humans, and may be particularly relevant for diseases, such as stroke, where gene‐environment interactions are likely to play an important role in pathogenesis.^45^ Substantial interindividual variation exists with respect to age and tissue type.^46^ However, an important emerging mechanism influencing methylation is local sequence content.^47^ Notably, recent studies have shown that GWAS findings from stroke‐relevant traits, such as blood pressure, are likely to act by influencing DNA methylation.^48^ This may be particularly relevant for SVS, in which environmental and other vascular risk factors, such as hypertension, are important and have been shown to interact with disease risk.^49^ We evaluated whether our associated SNP (rs12445022), or SNPs in close LD, influence methylation of nearby CpG sites. We found evidence from whole blood that the same genetic variation influences DNA methylation. SNPs in distant LD also influenced DNA methylation at a different probe (cg08031982) in the fetal brain. Further evidence comes from published studies in lung, breast, and kidney tissues,^31^ as well as in utero,^50^ all of which have shown that the genetic variation at the same 16q24.2 region influences methylation at the cg08031982 probe. Interestingly, the CpG sites influenced by the locus appear to differ by tissue, with different probes affected in whole blood compared to fetal brain. This might imply tissue‐specific functional consequences of the locus and therefore highlights the importance of performing follow‐up experiments in appropriate tissues. Based on the evidence presented here, we can only speculate on how genetic variation at the locus leads to increased risk of SVS. One hypothesis is that expression of *ZCCHC14*, or other proteins, is mediated through altered methylation of the probes identified. This might occur, in part, in response to environmental stimuli. Evaluating these hypotheses in a relevant tissue type will be an important future analysis to identify the causal mechanisms leading to SVS.

This study has limitations. Our results suggested that the association may be present in other ethnicities, but we had an insufficient number of cases to establish common risk conclusively. Follow‐up studies are therefore required in other ethnic groups. In addition, downstream functional experiments will be required to determine the consequences of the identified association. The mRNA expression and methylation analyses presented herein were constrained by available tissue types. Validation of the findings in more disease‐relevant tissue types, such as cerebral small vessels, therefore represent important follow‐up analyses, although obtaining such tissue in a state to allow mRNA studies is very challenging. We performed mRNA expression and methylation analyses using either the lead SNP (rs12445022) or three LD SNPs. The results should be interpreted with the limitation that we cannot be certain that any of these SNPs is the causal variant. Radiological confirmation of SVS in this study was performed using either computed tomography or MRI. Evidence shows that MRI is considerably more reliable at identifying SVS. Replication of the association in an MRI‐confirmed population may therefore provide a more‐accurate estimate of the effect of the locus on SVS risk. Similarly, interrogation of causative classification system definitions of SVS may provide further insights.^51^ Another method of interrogating the combined influence of age and genotype is by testing for an interaction. In this analysis, we were unable to do this because age was not available in some sets of controls (eg, WTCCC2).

In this large genome‐wide meta‐analysis using an age‐at‐onset informed approach, we have identified the first genome‐wide significant locus that is associated solely with SVS. Our findings, which point to subtle changes in gene expression and DNA methylation influencing disease risk, show that strategies that account for different liability across disease‐related covariates, such as age, can identify novel associations with disease.

## Author Contributions

M.T., R.M., C.M.L., B.B.W., and H.S.M. conceived and designed the study. M.T., B.B.W., and H.S.M. drafted the manuscript. M.T. and K.B.H. drew the figures. M.T., R.M., M.A.N., I.C., F.R., P.S., D.S., M.A.H.‐B., C.L.M.S., P.M.R., G.B., V.T., R.L., C.L., J.F.M., J.R., M.D., B.B.W., and H.S.M. contributed acquisition and analysis of METASTROKE data sets. H.X., L.H., M.F., C.J., J.F.M., B.D.M., S.J.K., M.D., B.B.W., J.J.‐C., J.W.C., R.S., A.S., R.L., A.L., O.M., R.P.G., R.L.S., T.R., K.R., D.K.A., J.A.J., O.R.B., S.W.‐S., J‐M.L., M.T., S.S.R., P.D.B., S.L.P., Q.W., P.F.M., D.W., C.D.A., and J.R. contributed acquisition and analysis of the NINDS‐SIGN data. J.R., D.W., C.L., and C.D.A. contributed acquisition and analysis of ICH data. G.C., S.D., L.J.L., S.S., J.C.B., and W.T.L., Jr., contributed acquisition and analysis of neuro‐CHARGE data. I.Y., T.D.S., J.T.B., E.H., and J.M. contributed acquisition and analysis of DNA methylation data. M.T., H.S.M., and N.S.R. contributed acquisition and analysis of WMH data. G.T., K.S., U.T., and S.G. contributed acquisition and analysis of deCODE data.

## Potential Conflicts of Interest

Authors whose affiliations are listed as deCODE/Amgen are employees of deCODE/ Amgen.

## Supporting information

Additional supporting information can be found in the online version of this article.

Supporting Information Table 1.

Supporting Information Table 2.

Supporting Information Table 3.

Supporting Information Table 4.
