# Supplementary material for: Genetic variation at 16q24.2 is associated with small vessel stroke
Source: Ann Neurol. 2017 Mar 25;81(3):383–94. doi: 10.1002/ana.24840 (PMC5366092; doi:10.1002/ana.24840)
Supplement: Supplementary file 1 — Supporting Information Table 1. [file ANA-81-383-s001.docx]

Supplementary Table 1 – Small Vessel Stroke association statistics for SNPs taken forward to Stage II

|  |  |  |  | Stage I |  |  |  |  | Stage II & III |  |  | Overall |  |
| --- | --- | --- | --- | --- | --- | --- | --- | --- | --- | --- | --- | --- | --- |
| SNP | CHR | BP | Allele1 | Allele2 | Freq1 | Zscore | P.value | Freq1 | Zscore | P.value | Freq1 | Zscore | P.value |
| rs12445022 | 16 | 87575332 | a | g | 0.3378 | 4.813 | 1.49E-06 | 0.3233 | 3.981 | 6.87E-05 | 0.3273 | 5.923 | 3.17E-09 |
| rs12920915 | 16 | 87577521 | t | c | 0.4041 | 4.255 | 2.10E-05 | 0.3999 | 3.353 | 0.0007993 | 0.4018 | 5.337 | 9.44E-08 |
| rs17879928 | 1 | 92160300 | a | g | 0.7982 | 4.603 | 4.17E-06 | 0.7951 | 2.430 | 0.01509 | 0.7897 | 4.495 | 6.95E-07 |
| rs11807260 | 1 | 92160761 | a | g | 0.2019 | -4.595 | 4.32E-06 | 0.2051 | -2.238 | 0.00466 | 0.2037 | -5.175 | 2.27E-07 |
| rs12444224 | 16 | 87580855 | t | c | 0.4035 | 4.149 | 3.34E-05 | 0.4008 | 3.224 | 0.001266 | 0.402 | 5.17 | 2.34E-07 |
| rs6693438 | 1 | 92163174 | a | g | 0.7978 | 4.583 | 4.58E-06 | 0.794 | 2.151 | 0.006036 | 0.7957 | 5.105 | 3.31E-07 |
| rs1961463 | 10 | 3506218 | a | g | 0.2904 | -4.651 | 3.30E-06 | 0.2891 | -2.356 | 0.01848 | 0.2897 | -4.86 | 1.17E-06 |
| rs2053889 | 10 | 3509300 | t | g | 0.2922 | -4.42 | 9.89E-06 | 0.2902 | -2.524 | 0.0116 | 0.2911 | -4.83 | 1.36E-06 |
| rs8073136 | 17 | 52654201 | t | c | 0.7574 | -4.285 | 1.83E-05 | 0.7576 | -2.594 | 0.0095 | 0.7575 | -4.792 | 1.65E-06 |
| rs7225084 | 17 | 52652493 | t | g | 0.7585 | -4.266 | 1.99E-05 | 0.759 | -2.513 | 0.01199 | 0.7588 | -4.719 | 2.37E-06 |
| rs115936795 | 17 | 2572778 | t | g | 0.0158 | 4.481 | 7.44E-06 | 0.0149 | 2.354 | 0.01856 | 0.0153 | 4.68 | 2.86E-06 |
| rs12244869 | 10 | 3499052 | c | g | 0.7037 | 4.529 | 5.94E-06 | 0.7032 | 2.184 | 0.02894 | 0.7034 | 4.651 | 3.31E-06 |
| rs138515134 | 17 | 2579590 | t | g | 0.9852 | -4.501 | 6.77E-06 | 0.9856 | -2.276 | 0.02284 | 0.9854 | -4.633 | 3.60E-06 |
| rs2439998 | 17 | 2537551 | t | c | 0.0189 | 4.47 | 7.81E-06 | 0.0181 | 2.188 | 0.02864 | 0.0185 | 4.599 | 4.24E-06 |
| rs73519263 | 16 | 27123478 | t | g | 0.9822 | -4.714 | 2.42E-06 | 0.9795 | -2.085 | 0.03709 | 0.9805 | -4.5 | 6.79E-06 |
| rs73519272 | 16 | 27124978 | t | g | 0.0178 | 4.657 | 3.21E-06 | 0.0206 | 2.056 | 0.03983 | 0.0196 | 4.442 | 8.90E-06 |
| rs896644 | 18 | 59247445 | a | t | 0.9107 | -4.564 | 5.01E-06 | 0.9107 | -1.801 | 0.07176 | 0.9107 | -4.389 | 1.14E-05 |
| rs1452583 | 18 | 59241941 | a | t | 0.0883 | 4.555 | 5.24E-06 | 0.0893 | 1.751 | 0.07989 | 0.0889 | 4.346 | 1.39E-05 |
| rs148398060 | 16 | 27105221 | a | g | 0.017 | 4.494 | 7.01E-06 | 0.0202 | 1.803 | 0.07147 | 0.0189 | 4.24 | 2.24E-05 |
| rs9827208 | 3 | 7908121 | a | g | 0.688 | -5.102 | 3.36E-07 | 0.6925 | -1.1 | 0.2713 | 0.6905 | -4.227 | 2.37E-05 |
| rs6567220 | 18 | 59241643 | a | g | 0.9098 | -4.665 | 3.08E-06 | 0.9076 | -1.475 | 0.1401 | 0.9086 | -4.214 | 2.51E-05 |
| rs2083833 | 17 | 52655010 | t | c | 0.192 | 4.353 | 1.35E-05 | 0.1892 | 1.742 | 0.08143 | 0.1904 | 4.204 | 2.62E-05 |
| rs7615995 | 3 | 7923100 | a | t | 0.3109 | 5.048 | 4.46E-07 | 0.3085 | 1.117 | 0.2641 | 0.3096 | 4.203 | 2.63E-05 |
| rs13386661 | 2 | 59883352 | t | c | 0.0196 | 4.61 | 4.03E-06 | 0.0189 | 1.528 | 0.1265 | 0.0192 | 4.153 | 3.29E-05 |
| rs72952365 | 2 | 59859190 | t | c | 0.0162 | 4.212 | 2.53E-05 | 0.0182 | 1.744 | 0.08115 | 0.0174 | 4.059 | 4.93E-05 |
| rs6737557 | 2 | 59885842 | t | c | 0.9834 | -4.177 | 2.95E-05 | 0.981 | -1.693 | 0.09036 | 0.982 | -3.998 | 6.39E-05 |
| rs17076342 | 3 | 44079629 | a | g | 0.8959 | -4.171 | 3.03E-05 | 0.9035 | -1.185 | 0.2361 | 0.9001 | -3.668 | 0.0002445 |
| rs9871447 | 3 | 44079944 | t | c | 0.103 | 4.11 | 3.96E-05 | 0.0976 | 1.234 | 0.2172 | 0.1 | 3.663 | 0.0002489 |
| rs9817280 | 3 | 44083722 | a | g | 0.0986 | 4.155 | 3.26E-05 | 0.0965 | 1.182 | 0.2371 | 0.0974 | 3.655 | 0.0002571 |
| rs373151 | 3 | 7927037 | a | g | 0.5832 | 4.992 | 5.97E-07 | 0.593 | 0.421 | 0.6738 | 0.5886 | 3.648 | 0.0002644 |
| rs2019441 | 18 | 23197880 | t | c | 0.9643 | -4.465 | 8.00E-06 | 0.9652 | -0.647 | 0.5178 | 0.9648 | -3.464 | 0.0005324 |
| rs870399 | 11 | 131456204 | t | c | 0.2958 | -4.114 | 3.88E-05 | 0.2894 | -0.902 | 0.3673 | 0.2922 | -3.403 | 0.0006656 |
| rs9356705 | 6 | 19677864 | t | c | 0.0358 | 4.859 | 1.18E-06 | 0.0337 | 0.334 | 0.7384 | 0.0346 | 3.375 | 0.0007389 |
| rs73395542 | 18 | 23192332 | a | g | 0.9639 | -4.336 | 1.45E-05 | 0.9649 | -0.534 | 0.593 | 0.9645 | -3.294 | 0.0009886 |
| rs4800650 | 18 | 23192273 | a | t | 0.9639 | -4.334 | 1.47E-05 | 0.9649 | -0.531 | 0.5953 | 0.9645 | -3.29 | 0.001002 |
| rs2190179 | 7 | 79045496 | a | c | 0.265 | 4.282 | 1.85E-05 | 0.273 | 0.514 | 0.6069 | 0.2694 | 3.243 | 0.001181 |
| rs10238315 | 7 | 79043013 | t | g | 0.2653 | 4.279 | 1.88E-05 | 0.2729 | 0.503 | 0.6152 | 0.2695 | 3.232 | 0.001229 |
| rs2288131 | 12 | 29683089 | t | c | 0.3092 | 4.347 | 1.38E-05 | 0.3124 | 0.391 | 0.696 | 0.311 | 3.194 | 0.001401 |
| rs299456 | 12 | 29695861 | a | g | 0.3155 | 4.246 | 2.17E-05 | 0.3198 | 0.277 | 0.782 | 0.3179 | 3.042 | 0.002348 |
| rs299457 | 12 | 29696329 | t | c | 0.3156 | 4.245 | 2.18E-05 | 0.3198 | 0.271 | 0.7866 | 0.3179 | 3.037 | 0.002388 |
| rs4730806 | 7 | 79030922 | a | c | 0.7351 | -4.103 | 4.08E-05 | 0.7269 | -0.305 | 0.7601 | 0.7306 | -2.968 | 0.002998 |
| rs60475239 | 2 | 219047216 | a | t | 0.5464 | -4.811 | 1.50E-06 | 0.3428 | 1.062 | 0.2884 | 0.4529 | -2.819 | 0.004813 |
| rs9368108 | 6 | 19692570 | a | t | 0.9555 | -4.864 | 1.15E-06 | 0.9604 | 0.43 | 0.6673 | 0.9584 | -2.792 | 0.005238 |
| rs72657608 | 6 | 19691522 | t | c | 0.0445 | 4.864 | 1.15E-06 | 0.0397 | -0.433 | 0.6647 | 0.0417 | 2.789 | 0.005281 |
| rs4937638 | 11 | 131456484 | a | t | 0.4282 | 4.219 | 2.46E-05 | 0.4153 | 0.221 | 0.825 | 0.4201 | 2.76 | 0.005779 |
| rs12363659 | 11 | 131456719 | a | g | 0.5718 | -4.211 | 2.54E-05 | 0.5847 | -0.215 | 0.8296 | 0.5799 | -2.751 | 0.005948 |
| rs12743454 | 1 | 208339738 | a | g | 0.6561 | 4.372 | 1.23E-05 | 0.6511 | -0.313 | 0.7544 | 0.6533 | 2.687 | 0.007203 |
| rs2590685 | 1 | 208339232 | a | g | 0.6553 | 4.316 | 1.59E-05 | 0.6493 | -0.287 | 0.7739 | 0.652 | 2.669 | 0.007609 |
| rs4674267 | 2 | 219046437 | t | c | 0.4322 | 4.894 | 9.86E-07 | 0.4468 | -1.073 | 0.2834 | 0.4403 | 2.471 | 0.01348 |
| rs11894169 | 2 | 219046846 | t | c | 0.4332 | 4.802 | 1.57E-06 | 0.4474 | -1.075 | 0.2823 | 0.4411 | 2.408 | 0.01605 |
| rs13181146 | 5 | 176128648 | a | t | 0.8334 | 4.329 | 1.50E-05 | 0.8192 | -0.735 | 0.4622 | 0.8252 | 2.247 | 0.02462 |
| rs2884531 | 5 | 176129392 | t | c | 0.1664 | -4.275 | 1.91E-05 | 0.1812 | 0.794 | 0.4273 | 0.175 | -2.168 | 0.03018 |
| rs9515350 | 13 | 111668229 | a | g | 0.869 | -3.552 | 0.0003818 | 0.8635 | 0.425 | 0.671 | 0.866 | -2.057 | 0.03971 |
| rs139728593 | 8 | 99641925 | a | c | 0.0122 | 4.741 | 2.13E-06 | 0.0109 | -1.455 | 0.1456 | 0.0115 | 2.023 | 0.04309 |
| rs11737790 | 4 | 120751664 | t | c | 0.337 | 4.304 | 1.68E-05 | 0.3274 | -1.17 | 0.242 | 0.3317 | 2.004 | 0.04503 |
| rs4868697 | 5 | 176129823 | c | g | 0.8232 | 4.196 | 2.72E-05 | 0.8054 | -0.997 | 0.3186 | 0.8129 | 1.962 | 0.04981 |
| rs6534157 | 4 | 120753056 | c | g | 0.6642 | -4.272 | 1.94E-05 | 0.6738 | 1.286 | 0.1983 | 0.6695 | -1.896 | 0.05791 |
| rs6858155 | 4 | 120754216 | t | g | 0.3358 | 4.27 | 1.96E-05 | 0.3266 | -1.306 | 0.1917 | 0.3307 | 1.88 | 0.06006 |
| rs10996826 | 10 | 67776227 | a | g | 0.5221 | -4.33 | 1.49E-05 | 0.5205 | 1.398 | 0.1621 | 0.5212 | -1.852 | 0.06406 |
| rs10822693 | 10 | 67776999 | c | g | 0.478 | 4.331 | 1.48E-05 | 0.4795 | -1.4 | 0.1614 | 0.4788 | 1.851 | 0.06419 |
| rs189950918 | 8 | 99535634 | c | g | 0.0117 | 4.342 | 1.41E-05 | 0.0103 | -1.36 | 0.1737 | 0.0109 | 1.832 | 0.06695 |
| rs7098171 | 10 | 67780663 | t | c | 0.5133 | -4.437 | 9.14E-06 | 0.518 | 1.527 | 0.1267 | 0.5159 | -1.827 | 0.06772 |
| rs6656221 | 1 | 208329618 | a | g | 0.6852 | 3.907 | 9.35E-05 | 0.687 | -1.164 | 0.2443 | 0.6862 | 1.743 | 0.08129 |
| rs67730808 | 13 | 111661710 | a | g | 0.1347 | 3.307 | 0.0009441 | 0.1362 | -0.691 | 0.4899 | 0.1355 | 1.695 | 0.09009 |
| rs146021580 | 3 | 121766140 | a | c | 0.0244 | 4.355 | 1.33E-05 | 0.0235 | -1.677 | 0.09346 | 0.0239 | 1.661 | 0.09672 |
| rs9515357 | 13 | 111683822 | t | c | 0.128 | 3.426 | 0.0006122 | 0.1217 | -0.889 | 0.3738 | 0.1245 | 1.627 | 0.1038 |
| rs35538837 | 3 | 121659202 | a | g | 0.0269 | 4.188 | 2.82E-05 | 0.0262 | -1.659 | 0.09718 | 0.0265 | 1.563 | 0.1181 |
| rs142283780 | 22 | 24334674 | t | c | 0.1523 | 4.075 | 4.59E-05 | 0.1538 | -1.161 | 0.2458 | 0.1532 | 1.559 | 0.1189 |
| rs118094794 | 8 | 99479692 | a | g | 0.9882 | -4.039 | 5.38E-05 | 0.9898 | 1.47 | 0.1415 | 0.9891 | -1.549 | 0.1213 |
| rs140004544 | 3 | 121689502 | t | c | 0.0281 | 4.245 | 2.19E-05 | 0.0273 | -1.761 | 0.07824 | 0.0277 | 1.525 | 0.1273 |
| rs4337774 | 4 | 67107733 | a | g | 0.0548 | 4.243 | 2.21E-05 | 0.054 | -1.633 | 0.1024 | 0.0543 | 1.508 | 0.1317 |
| rs11131636 | 4 | 67108352 | a | g | 0.0572 | 4.321 | 1.55E-05 | 0.0544 | -1.731 | 0.0834 | 0.0556 | 1.484 | 0.1379 |
| rs79215719 | 4 | 67106740 | c | g | 0.0544 | 4.288 | 1.80E-05 | 0.0523 | -1.734 | 0.08288 | 0.0532 | 1.46 | 0.1442 |
| rs7291499 | 22 | 24341101 | a | g | 0.1545 | 4.17 | 3.04E-05 | 0.1542 | -1.395 | 0.1631 | 0.1543 | 1.431 | 0.1524 |
| rs182268832 | 22 | 24334133 | t | g | 0.1456 | 4.062 | 4.88E-05 | 0.1484 | -1.371 | 0.1705 | 0.1474 | 1.384 | 0.1663 |

CHR, chromosome; BP, base position; Freq1, frequency of Allele1; Stage III results included for rs12445022 and rs17879928 only
