# Supplementary material for: Genetic variation at 16q24.2 is associated with small vessel stroke
Source: Ann Neurol. 2017 Mar 25;81(3):383–94. doi: 10.1002/ana.24840 (PMC5366092; doi:10.1002/ana.24840)
Supplement: Supplementary file 2 — Supporting Information Table 2. [file ANA-81-383-s002.docx]

Supplementary Table 2 – Large Artery Stroke association statistics for SNPs taken forward to Stage II

|  |  |  |  | Stage I |  |  |  |  | Stage II |  |  | Overall |  |
| --- | --- | --- | --- | --- | --- | --- | --- | --- | --- | --- | --- | --- | --- |
| SNP | CHR | BP | Allele1 | Allele2 | Freq1 | Zscore | P.value | Freq1 | Zscore | P.value | Freq1 | Zscore | P.value |
| rs71524263 | 7 | 19030278 | t | c | 0.1157 | 5.309 | 1.10E-07 | 0.1218 | 4.769 | 1.85E-06 | 0.1194 | 7.047 | 1.83E-12 |
| rs2107595 | 7 | 19049388 | a | g | 0.1614 | 5.541 | 3.02E-08 | 0.163 | 4.19 | 2.78E-05 | 0.1623 | 6.815 | 9.46E-12 |
| rs57301765 | 7 | 19052733 | a | g | 0.1634 | 5.502 | 3.77E-08 | 0.1669 | 4.134 | 3.56E-05 | 0.1654 | 6.747 | 1.51E-11 |
| rs12939005 | 17 | 52986484 | t | g | 0.2018 | 4.379 | 1.19E-05 | 0.1903 | 2.384 | 0.01712 | 0.1954 | 4.693 | 2.69E-06 |
| rs72749603 | 5 | 31178840 | a | c | 0.9636 | -4.865 | 1.14E-06 | 0.9628 | -1.943 | 0.05206 | 0.9632 | -4.687 | 2.77E-06 |
| rs72829469 | 17 | 52989920 | a | g | 0.2025 | 4.375 | 1.22E-05 | 0.1904 | 2.352 | 0.01868 | 0.1958 | 4.666 | 3.07E-06 |
| rs2332312 | 17 | 52996429 | a | g | 0.2033 | 4.394 | 1.11E-05 | 0.1927 | 2.202 | 0.02769 | 0.1974 | 4.567 | 4.95E-06 |
| rs62367577 | 5 | 86802658 | t | g | 0.8479 | -4.236 | 2.28E-05 | 0.8436 | -2.299 | 0.02152 | 0.8455 | -4.534 | 5.78E-06 |
| rs7342953 | 17 | 63245585 | t | c | 0.0316 | 4.722 | 2.33E-06 | 0.0298 | 1.83 | 0.0672 | 0.0306 | 4.508 | 6.54E-06 |
| rs59192960 | 5 | 31181789 | t | c | 0.0385 | 4.569 | 4.91E-06 | 0.0377 | 1.903 | 0.05708 | 0.0381 | 4.46 | 8.20E-06 |
| rs80160193 | 17 | 63243822 | a | t | 0.0316 | 4.642 | 3.45E-06 | 0.0299 | 1.787 | 0.07395 | 0.0307 | 4.422 | 9.76E-06 |
| rs76490029 | 17 | 63243674 | t | c | 0.0316 | 4.64 | 3.48E-06 | 0.0299 | 1.787 | 0.074 | 0.0307 | 4.421 | 9.84E-06 |
| rs11696561 | 20 | 17455185 | a | g | 0.2813 | -4.492 | 7.04E-06 | 0.2773 | -1.577 | 0.1149 | 0.2791 | -4.166 | 3.11E-05 |
| rs4239702 | 20 | 44749251 | t | c | 0.2749 | 4.652 | 3.29E-06 | 0.2867 | 1.423 | 0.1546 | 0.2815 | 4.157 | 3.22E-05 |
| rs55862206 | 4 | 129591442 | c | g | 0.9211 | -4.677 | 2.91E-06 | 0.9227 | -1.378 | 0.1681 | 0.922 | -4.14 | 3.47E-05 |
| rs56299512 | 4 | 129591445 | a | g | 0.9211 | -4.677 | 2.91E-06 | 0.9227 | -1.378 | 0.1681 | 0.922 | -4.14 | 3.47E-05 |
| rs10811652 | 9 | 22077085 | a | c | 0.5057 | -4.419 | 9.93E-06 | 0.4967 | -1.567 | 0.1171 | 0.5007 | -4.109 | 3.97E-05 |
| rs72783748 | 5 | 86863605 | a | g | 0.7635 | -4.319 | 1.57E-05 | 0.7485 | -1.638 | 0.1014 | 0.7551 | -4.096 | 4.20E-05 |
| rs10757271 | 9 | 22076795 | a | g | 0.5051 | -4.37 | 1.24E-05 | 0.4962 | -1.586 | 0.1128 | 0.5001 | -4.091 | 4.30E-05 |
| rs34562050 | 4 | 129594713 | c | g | 0.08 | 4.677 | 2.91E-06 | 0.0789 | 1.224 | 0.221 | 0.0794 | 4.025 | 5.69E-05 |
| rs11607832 | 11 | 102544684 | t | c | 0.9299 | -4.008 | 6.13E-05 | 0.9332 | -1.761 | 0.0783 | 0.9317 | -3.981 | 6.88E-05 |
| rs9632884 | 9 | 22072301 | c | g | 0.4918 | 4.389 | 1.14E-05 | 0.5007 | 1.241 | 0.2145 | 0.4968 | 3.846 | 0.00012 |
| rs11905301 | 20 | 24531347 | t | c | 0.4669 | 4.274 | 1.92E-05 | 0.5327 | 1.404 | 0.1603 | 0.5054 | 3.829 | 0.0001289 |
| rs7720392 | 5 | 31170385 | t | c | 0.9661 | -4.074 | 4.63E-05 | 0.9668 | -1.451 | 0.1467 | 0.9665 | -3.793 | 0.0001486 |
| rs4810485 | 20 | 44747947 | t | g | 0.2506 | 4.092 | 4.29E-05 | 0.2608 | 1.413 | 0.1576 | 0.2563 | 3.777 | 0.0001587 |
| rs12635174 | 3 | 117008209 | t | c | 0.7228 | 4.45 | 8.60E-06 | 0.7307 | 1.08 | 0.28 | 0.7272 | 3.767 | 0.0001654 |
| rs11696580 | 20 | 17455276 | t | g | 0.1926 | -4.222 | 2.42E-05 | 0.198 | -1.247 | 0.2122 | 0.1956 | -3.74 | 0.0001838 |
| rs1883832 | 20 | 44746982 | t | c | 0.2507 | 4.039 | 5.38E-05 | 0.2607 | 1.405 | 0.16 | 0.2563 | 3.736 | 0.0001872 |
| rs74725770 | 7 | 93946272 | a | g | 0.989 | -4.857 | 1.19E-06 | 0.989 | -0.903 | 0.3665 | 0.989 | -3.716 | 0.0002021 |
| rs10049229 | 3 | 117001187 | a | g | 0.2775 | -4.45 | 8.58E-06 | 0.2703 | -0.962 | 0.3359 | 0.2735 | -3.679 | 0.0002342 |
| rs6075215 | 20 | 17454302 | a | g | 0.1931 | -4.225 | 2.40E-05 | 0.1983 | -1.142 | 0.2534 | 0.196 | -3.663 | 0.0002492 |
| rs13092499 | 3 | 117009819 | a | c | 0.7229 | 4.45 | 8.58E-06 | 0.73 | 0.863 | 0.388 | 0.7269 | 3.605 | 0.0003122 |
| rs13039850 | 20 | 24547144 | t | c | 0.3574 | -4.545 | 5.51E-06 | 0.348 | -0.646 | 0.5181 | 0.3522 | -3.506 | 0.0004554 |
| rs1561734 | 5 | 86826750 | a | g | 0.2109 | 4.298 | 1.73E-05 | 0.2253 | 0.839 | 0.4015 | 0.2189 | 3.485 | 0.0004913 |
| rs75684303 | 7 | 93878978 | a | g | 0.011 | 4.704 | 2.55E-06 | 0.008 | 0.773 | 0.4398 | 0.0091 | 3.442 | 0.0005773 |
| rs11159150 | 14 | 76162001 | a | g | 0.3084 | -4.34 | 1.43E-05 | 0.3038 | -0.646 | 0.5185 | 0.3058 | -3.369 | 0.0007545 |
| rs1005224 | 14 | 76173860 | a | t | 0.6902 | 4.286 | 1.82E-05 | 0.6912 | 0.683 | 0.4944 | 0.6908 | 3.361 | 0.0007756 |
| rs727409 | 20 | 24557527 | t | c | 0.3202 | -4.252 | 2.12E-05 | 0.3164 | -0.692 | 0.4889 | 0.3181 | -3.345 | 0.0008222 |
| rs662558 | 11 | 102718695 | t | c | 0.8213 | -3.524 | 0.0004255 | 0.8117 | -1.321 | 0.1865 | 0.8159 | -3.33 | 0.0008671 |
| rs2660040 | 10 | 68275581 | a | g | 0.294 | 4.797 | 1.61E-06 | 0.2961 | 0.142 | 0.8872 | 0.2952 | 3.297 | 0.000978 |
| rs8073683 | 17 | 17555596 | t | c | 0.474 | -4.525 | 6.05E-06 | 0.4749 | -0.327 | 0.7436 | 0.4745 | -3.254 | 0.001137 |
| rs1522966 | 3 | 34843726 | a | t | 0.5229 | 4.594 | 4.36E-06 | 0.5211 | 0.227 | 0.8206 | 0.5219 | 3.225 | 0.001259 |
| rs118001792 | 11 | 41905979 | a | c | 0.0136 | 4.391 | 1.13E-05 | 0.0143 | 0.32 | 0.7492 | 0.014 | 3.16 | 0.00158 |
| rs586701 | 11 | 102724730 | t | g | 0.8236 | -3.194 | 0.001405 | 0.8134 | -1.372 | 0.1701 | 0.8179 | -3.149 | 0.001639 |
| rs61103969 | 11 | 41897789 | a | g | 0.0136 | 4.526 | 6.00E-06 | 0.0145 | 0.174 | 0.8622 | 0.0141 | 3.141 | 0.001685 |
| rs525673 | 3 | 34848226 | t | c | 0.523 | 4.636 | 3.56E-06 | 0.5227 | 0.063 | 0.95 | 0.5228 | 3.131 | 0.001744 |
| rs7893676 | 10 | 68270632 | c | g | 0.2923 | 4.152 | 3.30E-05 | 0.2863 | 0.483 | 0.629 | 0.289 | 3.123 | 0.001791 |
| rs12258032 | 10 | 68271859 | t | c | 0.7076 | -4.138 | 3.50E-05 | 0.7137 | -0.485 | 0.6275 | 0.711 | -3.115 | 0.001838 |
| rs1916227 | 3 | 34847097 | a | g | 0.4774 | -4.58 | 4.64E-06 | 0.4776 | -0.069 | 0.9454 | 0.4775 | -3.098 | 0.001947 |
| rs11626058 | 14 | 76115445 | a | c | 0.3724 | -4.253 | 2.11E-05 | 0.3734 | -0.346 | 0.7296 | 0.373 | -3.087 | 0.002021 |
| rs58980959 | 11 | 41903641 | c | g | 0.0136 | 4.387 | 1.15E-05 | 0.0145 | 0.194 | 0.8461 | 0.0141 | 3.063 | 0.00219 |
| rs7164538 | 15 | 60155804 | a | g | 0.9679 | 4.824 | 1.41E-06 | 0.9714 | -0.337 | 0.7361 | 0.9699 | 2.957 | 0.003103 |
| rs7164565 | 15 | 60155858 | a | g | 0.9679 | 4.823 | 1.41E-06 | 0.9714 | -0.338 | 0.7356 | 0.9699 | 2.957 | 0.00311 |
| rs191633333 | 7 | 93746175 | a | c | 0.0112 | 4.804 | 1.56E-06 | 0.011 | -0.065 | 0.9484 | 0.0111 | 2.872 | 0.004074 |
| rs12414028 | 10 | 104957629 | a | t | 0.0924 | -4.102 | 4.10E-05 | 0.0959 | -0.181 | 0.856 | 0.0944 | -2.864 | 0.004181 |
| rs2099580 | 15 | 60157890 | t | g | 0.9683 | 4.852 | 1.22E-06 | 0.9718 | -0.449 | 0.6535 | 0.9703 | 2.861 | 0.004218 |
| rs16870743 | 5 | 73016810 | a | g | 0.7238 | -4.465 | 8.01E-06 | 0.7232 | 0.168 | 0.8664 | 0.7235 | -2.845 | 0.004447 |
| rs644993 | 11 | 97541637 | a | g | 0.1229 | 4.371 | 1.24E-05 | 0.1276 | -0.138 | 0.8906 | 0.1255 | 2.805 | 0.005027 |
| rs79472290 | 4 | 156589785 | t | c | 0.0149 | 4.065 | 4.80E-05 | 0.0152 | 0.384 | 0.701 | 0.0151 | 2.797 | 0.005153 |
| rs680900 | 11 | 97538429 | a | g | 0.127 | 4.419 | 9.91E-06 | 0.1336 | -0.231 | 0.8176 | 0.1307 | 2.768 | 0.005646 |
| rs59216006 | 5 | 73001469 | a | g | 0.7174 | -4.378 | 1.20E-05 | 0.7156 | 0.21 | 0.8338 | 0.7164 | -2.756 | 0.005856 |
| rs17400036 | 6 | 69707211 | t | g | 0.9298 | -4.285 | 1.83E-05 | 0.9294 | 0.133 | 0.8939 | 0.9296 | -2.751 | 0.005944 |
| rs56120605 | 6 | 69714649 | a | g | 0.9299 | -4.32 | 1.56E-05 | 0.9304 | 0.174 | 0.8622 | 0.9302 | -2.744 | 0.006072 |
| rs6878052 | 5 | 72999965 | a | g | 0.2824 | 4.371 | 1.24E-05 | 0.2842 | -0.229 | 0.8188 | 0.2834 | 2.737 | 0.006202 |
| rs11191548 | 10 | 104846178 | t | c | 0.919 | 3.734 | 0.0001888 | 0.9099 | 0.297 | 0.7662 | 0.9139 | 2.706 | 0.006816 |
| rs9901291 | 17 | 17541750 | a | g | 0.4376 | 4.577 | 4.73E-06 | 0.447 | -0.482 | 0.6295 | 0.4428 | 2.684 | 0.007267 |
| rs597928 | 11 | 97545967 | a | c | 0.1251 | 4.444 | 8.85E-06 | 0.1282 | -0.375 | 0.708 | 0.1268 | 2.676 | 0.007442 |
| rs3740390 | 10 | 104638480 | t | c | 0.0841 | -3.774 | 0.0001605 | 0.092 | 0.004 | 0.9965 | 0.0885 | -2.507 | 0.01216 |
| rs12972736 | 19 | 6656777 | t | c | 0.2588 | -4.538 | 5.67E-06 | 0.2626 | 0.702 | 0.4829 | 0.261 | -2.389 | 0.01688 |
| rs12977994 | 19 | 6655261 | t | c | 0.2543 | -4.307 | 1.66E-05 | 0.2564 | 0.742 | 0.4579 | 0.2555 | -2.209 | 0.02717 |
| rs56111161 | 1 | 204695345 | t | c | 0.014 | 4.945 | 7.63E-07 | 0.0133 | -1.322 | 0.1863 | 0.0136 | 2.177 | 0.02946 |
| rs11240252 | 1 | 204691325 | a | g | 0.0143 | 4.98 | 6.35E-07 | 0.0138 | -1.703 | 0.08851 | 0.014 | 1.909 | 0.05632 |
| rs55878455 | 6 | 69639391 | t | c | 0.9259 | -3.878 | 0.0001054 | 0.9211 | 0.824 | 0.4097 | 0.9231 | -1.877 | 0.06059 |
| rs3738157 | 1 | 204684426 | a | g | 0.0119 | 4.941 | 7.76E-07 | 0.012 | -1.653 | 0.09839 | 0.012 | 1.818 | 0.06912 |
| rs62066209 | 17 | 17520784 | t | c | 0.388 | 4.531 | 5.88E-06 | 0.3868 | -1.609 | 0.1077 | 0.3873 | 1.813 | 0.06984 |
| rs12608923 | 19 | 6659855 | a | g | 0.2464 | -4.435 | 9.22E-06 | 0.2482 | 1.191 | 0.2337 | 0.2475 | -1.724 | 0.08466 |

CHR, chromosome; BP, base position; Freq1, frequency of Allele1
