# Supplementary material for: Genetic variation at 16q24.2 is associated with small vessel stroke
Source: Ann Neurol. 2017 Mar 25;81(3):383–94. doi: 10.1002/ana.24840 (PMC5366092; doi:10.1002/ana.24840)
Supplement: Supplementary file 3 — Supporting Information Table 3. [file ANA-81-383-s003.docx]

Supplementary Table 3 – Cardioembolic Stroke association statistics for SNPs taken forward to Stage II

|  |  |  |  | Stage I |  |  |  |  | Stage II & III |  |  | Overall |  |
| --- | --- | --- | --- | --- | --- | --- | --- | --- | --- | --- | --- | --- | --- |
| SNP | CHR | BP | Allele1 | Allele2 | Freq1 | Zscore | P.value | Freq1 | Zscore | P.value | Freq1 | Zscore | P.value |
| rs192172299 | 4 | 111681501 | t | g | 0.2148 | 5.994 | 2.05E-09 | 0.2217 | 6.832 | 8.39E-12 | 0.2187 | 9.088 | 1.01E-19 |
| rs2466455 | 4 | 111685615 | t | c | 0.7979 | -5.725 | 1.04E-08 | 0.7763 | -7.052 | 1.76E-12 | 0.7858 | -9.076 | 1.13E-19 |
| rs2634074 | 4 | 111677041 | a | t | 0.7975 | -5.739 | 9.55E-09 | 0.7759 | -7.018 | 2.25E-12 | 0.7854 | -9.059 | 1.32E-19 |
| rs879324 | 16 | 73068678 | a | g | 0.1721 | 5.026 | 5.02E-07 | 0.1681 | 3.371 | 0.0007501 | 0.1699 | 5.853 | 4.83E-09 |
| rs12932445 | 16 | 73069888 | t | c | 0.8278 | -4.866 | 1.14E-06 | 0.8313 | -3.293 | 0.0009929 | 0.8298 | -5.689 | 1.28E-08 |
| rs4499262 | 16 | 73059159 | a | c | 0.168 | 4.893 | 9.94E-07 | 0.165 | 3.043 | 0.002345 | 0.1663 | 5.519 | 3.40E-08 |
| rs8134198 | 21 | 45369535 | t | g | 0.3246 | 4.717 | 2.40E-06 | 0.3302 | 2.308 | 0.02102 | 0.3335 | 4.402 | 1.07E-05 |
| rs139223992 | 5 | 31110574 | t | c | 0.9116 | -4.593 | 4.37E-06 | 0.9115 | -2.319 | 0.02037 | 0.9115 | -4.779 | 1.77E-06 |
| rs72747745 | 5 | 31111931 | a | g | 0.0879 | 4.498 | 6.87E-06 | 0.088 | 2.372 | 0.01771 | 0.088 | 4.755 | 1.99E-06 |
| rs183560286 | 6 | 105692807 | a | g | 0.0769 | 4.254 | 2.10E-05 | 0.0719 | 2.64 | 0.008287 | 0.0739 | 4.721 | 2.35E-06 |
| rs4941428 | 13 | 43008177 | a | g | 0.2564 | 4.269 | 1.96E-05 | 0.2494 | 2.52 | 0.01172 | 0.2525 | 4.715 | 2.42E-06 |
| rs11740741 | 5 | 31114581 | t | c | 0.0897 | 4.573 | 4.81E-06 | 0.0888 | 2.198 | 0.02792 | 0.0892 | 4.675 | 2.94E-06 |
| rs7100632 | 10 | 134653951 | t | c | 0.5884 | -4.463 | 8.08E-06 | 0.6015 | -2.287 | 0.02218 | 0.5958 | -4.669 | 3.03E-06 |
| rs17065682 | 6 | 105704149 | t | g | 0.9235 | -4.091 | 4.29E-05 | 0.9269 | -2.683 | 0.007303 | 0.9256 | -4.653 | 3.28E-06 |
| rs17065694 | 6 | 105704658 | t | c | 0.0766 | 4.101 | 4.11E-05 | 0.073 | 2.648 | 0.008106 | 0.0744 | 4.631 | 3.63E-06 |
| rs3750580 | 10 | 134650440 | t | c | 0.6018 | -4.296 | 1.74E-05 | 0.6147 | -2.367 | 0.01794 | 0.609 | -4.618 | 3.88E-06 |
| rs4880434 | 10 | 134648569 | a | g | 0.5867 | -4.424 | 9.69E-06 | 0.6007 | -2.155 | 0.03113 | 0.5946 | -4.544 | 5.52E-06 |
| rs2147160 | 13 | 43021639 | a | c | 0.7445 | -4.036 | 5.43E-05 | 0.7516 | -2.491 | 0.01275 | 0.7485 | -4.539 | 5.66E-06 |
| rs1351832 | 13 | 43057549 | t | c | 0.255 | 4.064 | 4.82E-05 | 0.2483 | 2.417 | 0.01564 | 0.2512 | 4.502 | 6.73E-06 |
| rs11868827 | 17 | 69773732 | t | c | 0.1269 | -4.4 | 1.08E-05 | 0.1239 | -1.94 | 0.05237 | 0.1252 | -4.367 | 1.26E-05 |
| rs552148 | 9 | 136153481 | t | c | 0.2482 | -4.133 | 3.59E-05 | 0.2414 | -2.126 | 0.03353 | 0.2444 | -4.329 | 1.50E-05 |
| rs663367 | 9 | 136153451 | a | g | 0.2481 | -4.125 | 3.71E-05 | 0.2413 | -2.117 | 0.03428 | 0.2443 | -4.317 | 1.58E-05 |
| rs12453161 | 17 | 69752498 | a | g | 0.1291 | -4.431 | 9.36E-06 | 0.1252 | -1.84 | 0.06581 | 0.1269 | -4.312 | 1.62E-05 |
| rs500428 | 9 | 136155343 | a | g | 0.2483 | -4.115 | 3.88E-05 | 0.2415 | -2.11 | 0.03483 | 0.2445 | -4.305 | 1.67E-05 |
| rs1953522 | 1 | 216837241 | a | c | 0.3773 | -4.493 | 7.04E-06 | 0.3647 | -1.697 | 0.08962 | 0.3702 | -4.246 | 2.18E-05 |
| rs7252834 | 19 | 16075211 | a | g | 0.7727 | -4.205 | 2.61E-05 | 0.7502 | -1.774 | 0.07608 | 0.7601 | -4.113 | 3.90E-05 |
| rs73226947 | 21 | 45363522 | t | g | 0.3552 | 3.848 | 0.0001189 | 0.3598 | 2.042 | 0.04116 | 0.3578 | 4.065 | 4.79E-05 |
| rs2838444 | 21 | 45360862 | a | g | 0.3507 | 3.898 | 9.70E-05 | 0.3525 | 1.982 | 0.04745 | 0.3517 | 4.053 | 5.06E-05 |
| rs7252929 | 19 | 16075678 | t | c | 0.2274 | 4.257 | 2.07E-05 | 0.2493 | 1.627 | 0.1037 | 0.2397 | 4.038 | 5.40E-05 |
| rs1594692 | 19 | 16075015 | t | c | 0.7724 | -4.182 | 2.90E-05 | 0.7495 | -1.673 | 0.09437 | 0.7595 | -4.022 | 5.78E-05 |
| rs10958962 | 9 | 10229902 | t | c | 0.7405 | -4.381 | 1.18E-05 | 0.7464 | -1.451 | 0.1469 | 0.7438 | -3.987 | 6.69E-05 |
| rs2429937 | 17 | 69717560 | a | g | 0.1482 | -4.397 | 1.10E-05 | 0.1462 | -1.356 | 0.1752 | 0.1471 | -3.927 | 8.62E-05 |
| rs922982 | 9 | 84348195 | c | g | 0.4474 | 4.319 | 1.57E-05 | 0.4505 | 1.342 | 0.1796 | 0.4491 | 3.865 | 0.0001112 |
| rs4434691 | 9 | 84348762 | a | c | 0.4475 | 4.304 | 1.67E-05 | 0.4505 | 1.344 | 0.1788 | 0.4492 | 3.857 | 0.0001148 |
| rs7380303 | 5 | 168722937 | t | c | 0.7424 | -5.203 | 1.96E-07 | 0.7314 | -0.406 | 0.685 | 0.7362 | -3.748 | 0.0001783 |
| rs4242184 | 5 | 168726086 | a | g | 0.7436 | -5.09 | 3.58E-07 | 0.7328 | -0.466 | 0.6415 | 0.7375 | -3.718 | 0.0002007 |
| rs1455181 | 9 | 3774843 | a | c | 0.4018 | 4.726 | 2.29E-06 | 0.4009 | 0.712 | 0.4767 | 0.4013 | 3.661 | 0.0002509 |
| rs10061289 | 5 | 168718340 | c | g | 0.7666 | -5.142 | 2.71E-07 | 0.7587 | -0.337 | 0.7362 | 0.7622 | -3.656 | 0.0002559 |
| rs80129168 | 5 | 56481753 | a | g | 0.1082 | -4.049 | 5.14E-05 | 0.1057 | -1.227 | 0.2198 | 0.1068 | -3.583 | 0.0003403 |
| rs1997571 | 7 | 116198621 | a | g | 0.5916 | 4.2 | 2.67E-05 | 0.5846 | 1.033 | 0.3016 | 0.5877 | 3.554 | 0.0003789 |
| rs1997572 | 7 | 116198828 | a | g | 0.4084 | -4.2 | 2.67E-05 | 0.4154 | -1.033 | 0.3017 | 0.4123 | -3.554 | 0.0003794 |
| rs10867804 | 9 | 84341050 | a | c | 0.4322 | 4.326 | 1.52E-05 | 0.4379 | 0.903 | 0.3666 | 0.4354 | 3.54 | 0.0003999 |
| rs13361596 | 5 | 56540855 | t | c | 0.1088 | -4.007 | 6.15E-05 | 0.1078 | -1.207 | 0.2272 | 0.1082 | -3.54 | 4.00E-04 |
| rs3807989 | 7 | 116186241 | a | g | 0.4068 | -4.09 | 4.32E-05 | 0.4149 | -1.064 | 0.2875 | 0.4114 | -3.504 | 0.0004579 |
| rs2065070 | 9 | 10222790 | c | g | 0.7354 | -4.117 | 3.83E-05 | 0.7412 | -0.986 | 0.3239 | 0.7387 | -3.465 | 0.0005308 |
| rs12698965 | 7 | 70441601 | t | c | 0.6205 | 4.433 | 9.31E-06 | 0.6186 | 1.009 | 0.3128 | 0.6193 | 3.461 | 0.0005382 |
| rs3786778 | 19 | 48285809 | t | c | 0.9559 | -4.468 | 7.90E-06 | 0.9508 | -0.661 | 0.5088 | 0.953 | -3.453 | 0.0005551 |
| rs12983568 | 19 | 48272855 | t | c | 0.9551 | -4.495 | 6.96E-06 | 0.9528 | -0.63 | 0.5284 | 0.9538 | -3.448 | 0.0005651 |
| rs10269258 | 7 | 70440091 | t | c | 0.3795 | -4.438 | 9.06E-06 | 0.3817 | -0.982 | 0.326 | 0.3809 | -3.443 | 0.0005758 |
| rs6460609 | 7 | 70437060 | t | c | 0.6203 | 4.394 | 1.11E-05 | 0.6182 | 0.965 | 0.3344 | 0.619 | 3.403 | 0.0006674 |
| rs10471998 | 5 | 56447742 | a | g | 0.8859 | 4.458 | 8.26E-06 | 0.8908 | 0.911 | 0.3621 | 0.8891 | 3.366 | 0.0007637 |
| rs75781828 | 3 | 6994624 | t | c | 0.1182 | -4.347 | 1.38E-05 | 0.1123 | -0.548 | 0.5837 | 0.1149 | -3.288 | 0.001008 |
| rs12973532 | 19 | 48264879 | a | g | 0.9346 | -4.432 | 9.36E-06 | 0.9295 | -0.454 | 0.6501 | 0.9317 | -3.273 | 0.001063 |
| rs11071630 | 15 | 62098001 | t | c | 0.3935 | 4.466 | 7.98E-06 | 0.3968 | 0.421 | 0.6736 | 0.3954 | 3.272 | 0.001069 |
| rs10809038 | 9 | 10229301 | t | c | 0.8813 | -3.966 | 7.32E-05 | 0.8788 | -0.83 | 0.4067 | 0.8799 | -3.247 | 0.001167 |
| rs1472433 | 10 | 4661676 | a | g | 0.2765 | 4.461 | 8.18E-06 | 0.2749 | 0.388 | 0.6982 | 0.2756 | 3.243 | 0.001182 |
| rs10973456 | 9 | 3774359 | t | c | 0.5761 | -4.452 | 8.52E-06 | 0.5761 | -0.367 | 0.7139 | 0.5761 | -3.222 | 0.001275 |
| rs7910959 | 10 | 4646222 | a | t | 0.2755 | 4.466 | 7.97E-06 | 0.2743 | 0.354 | 0.7232 | 0.2748 | 3.222 | 0.001275 |
| rs149397829 | 3 | 6991641 | t | c | 0.1161 | -4.267 | 1.98E-05 | 0.1121 | -0.521 | 0.6021 | 0.1139 | -3.215 | 0.001303 |
| rs1021632 | 10 | 4652819 | t | g | 0.2764 | 4.451 | 8.54E-06 | 0.2753 | 0.357 | 0.721 | 0.2758 | 3.214 | 0.001309 |
| rs116972146 | 13 | 69632241 | t | c | 0.9641 | -4.219 | 2.46E-05 | 0.9659 | -0.562 | 0.5743 | 0.9651 | -3.214 | 0.001311 |
| rs13379934 | 15 | 62096931 | a | t | 0.7639 | -3.906 | 9.37E-05 | 0.7501 | -0.771 | 0.441 | 0.7561 | -3.163 | 0.00156 |
| rs181957491 | 13 | 69635572 | a | g | 0.9672 | -4.11 | 3.95E-05 | 0.9682 | -0.543 | 0.5869 | 0.9678 | -3.128 | 0.00176 |
| rs2163735 | 3 | 6995426 | a | g | 0.116 | -4.171 | 3.04E-05 | 0.1117 | -0.434 | 0.6643 | 0.1136 | -3.086 | 0.00203 |
| rs1268153 | 6 | 109035696 | a | g | 0.0324 | 4.286 | 1.82E-05 | 0.0319 | 0.512 | 0.6086 | 0.0321 | 3.081 | 0.002062 |
| rs34716044 | 15 | 62097134 | t | g | 0.762 | -3.767 | 0.0001655 | 0.7487 | -0.729 | 0.4658 | 0.7545 | -3.04 | 0.002367 |
| rs6901124 | 6 | 109035704 | t | c | 0.0314 | 4.092 | 4.27E-05 | 0.0299 | 0.584 | 0.5594 | 0.0305 | 3.016 | 0.002562 |
| rs78031559 | 13 | 69621457 | t | g | 0.9607 | -4.412 | 1.02E-05 | 0.9612 | 0.056 | 0.9553 | 0.961 | -2.878 | 0.003996 |
| rs11699273 | 20 | 58257735 | a | g | 0.0466 | 4.238 | 2.25E-05 | 0.0463 | 0.056 | 0.9553 | 0.0464 | 2.847 | 0.00441 |
| rs10814589 | 9 | 3772674 | a | g | 0.3173 | -2.646 | 0.008143 | 0.3204 | -1.436 | 0.1511 | 0.319 | -2.828 | 0.00469 |
| rs77749998 | 20 | 58259307 | t | c | 0.0418 | 4.193 | 2.76E-05 | 0.0438 | -0.272 | 0.7859 | 0.0429 | 2.572 | 0.01012 |
| rs11696365 | 20 | 58255101 | t | c | 0.0415 | 4.024 | 5.72E-05 | 0.0438 | -0.295 | 0.7677 | 0.0428 | 2.442 | 0.0146 |
| rs9384677 | 6 | 108863432 | a | c | 0.0398 | 3.86 | 0.0001133 | 0.042 | -0.278 | 0.7812 | 0.041 | 2.347 | 0.01893 |
| rs11847901 | 14 | 26862055 | a | g | 0.016 | 4.279 | 1.87E-05 | 0.0179 | -0.519 | 0.6035 | 0.0172 | 2.272 | 0.02306 |
| rs12566058 | 1 | 216182811 | a | g | 0.5483 | -4.216 | 2.48E-05 | 0.5329 | 0.7 | 0.4838 | 0.5396 | -2.266 | 0.02345 |
| rs11120712 | 1 | 216183467 | a | g | 0.465 | 4.296 | 1.74E-05 | 0.473 | -0.78 | 0.4353 | 0.4695 | 2.259 | 0.02391 |
| rs79205855 | 14 | 26855727 | c | g | 0.983 | -4.021 | 5.80E-05 | 0.9806 | 0.407 | 0.6841 | 0.9815 | -2.199 | 0.02791 |
| rs11845727 | 14 | 26850849 | t | c | 0.9834 | -3.945 | 7.99E-05 | 0.9814 | 0.418 | 0.676 | 0.9822 | -2.142 | 0.03218 |
| rs142258091 | 1 | 37772341 | a | t | 0.9807 | -4.762 | 1.92E-06 | 0.9837 | 1.405 | 0.16 | 0.9824 | -2.064 | 0.03905 |
| rs142632566 | 1 | 37747395 | t | c | 0.9807 | -4.814 | 1.48E-06 | 0.9838 | 1.486 | 0.1373 | 0.9825 | -2.037 | 0.04169 |
| rs141334771 | 1 | 37737497 | a | g | 0.0191 | 4.781 | 1.74E-06 | 0.0161 | -1.525 | 0.1273 | 0.0174 | 1.986 | 0.04704 |

CHR, chromosome; BP, base position; Freq1, frequency of Allele1; Stage III results included for rs8134198 only
