# Supplementary material for: Genetic variation at 16q24.2 is associated with small vessel stroke
Source: Ann Neurol. 2017 Mar 25;81(3):383–94. doi: 10.1002/ana.24840 (PMC5366092; doi:10.1002/ana.24840)
Supplement: Supplementary file 4 — Supporting Information Table 4. [file ANA-81-383-s004.docx]

Supplementary Table 4 – All Ischaemic Stroke association statistics for SNPs taken forward to Stage II

|  |  |  |  | Stage I |  |  |  |  | Stage II |  |  | Overall |  |
| --- | --- | --- | --- | --- | --- | --- | --- | --- | --- | --- | --- | --- | --- |
| SNP | CHR | BP | Allele1 | Allele2 | Freq1 | Zscore | P.value | Freq1 | Zscore | P.value | Freq1 | Zscore | P.value |
| rs11513729 | 12 | 112273499 | t | c | 0.4412 | 4.712 | 2.46E-06 | 0.4394 | 4.693 | 2.69E-06 | 0.4402 | 6.63 | 3.35E-11 |
| rs635634 | 9 | 136155000 | t | c | 0.1936 | 4.48 | 7.47E-06 | 0.2077 | 3.818 | 0.0001348 | 0.2009 | 5.86 | 4.64E-09 |
| rs532436 | 9 | 136149830 | a | g | 0.1928 | 4.435 | 9.19E-06 | 0.2078 | 3.86 | 0.0001132 | 0.2005 | 5.859 | 4.65E-09 |
| rs507666 | 9 | 136149399 | a | g | 0.1928 | 4.433 | 9.29E-06 | 0.2078 | 3.862 | 0.0001124 | 0.2005 | 5.859 | 4.66E-09 |
| rs58799304 | 1 | 156080444 | a | t | 0.2187 | -5.05 | 4.42E-07 | 0.2348 | -1.684 | 0.09225 | 0.2275 | -4.644 | 3.42E-06 |
| rs12035615 | 1 | 156055344 | t | c | 0.2231 | -4.767 | 1.87E-06 | 0.2227 | -1.753 | 0.07954 | 0.2229 | -4.579 | 4.68E-06 |
| rs1440410 | 4 | 144158309 | a | t | 0.5364 | -4.481 | 7.43E-06 | 0.5306 | -2.024 | 0.04296 | 0.5334 | -4.573 | 4.80E-06 |
| rs3814314 | 1 | 156045183 | a | t | 0.2263 | -4.774 | 1.81E-06 | 0.2262 | -1.739 | 0.08197 | 0.2262 | -4.573 | 4.80E-06 |
| rs181541997 | 6 | 47107964 | a | c | 0.0251 | 4.577 | 4.71E-06 | 0.0224 | 1.816 | 0.06939 | 0.0237 | 4.491 | 7.09E-06 |
| rs4322086 | 9 | 85787718 | a | g | 0.5302 | 4.382 | 1.18E-05 | 0.5399 | 2.003 | 0.04522 | 0.5352 | 4.489 | 7.16E-06 |
| rs761521 | 6 | 47112002 | t | c | 0.0252 | 4.618 | 3.88E-06 | 0.0224 | 1.746 | 0.08086 | 0.0238 | 4.469 | 7.86E-06 |
| rs4618266 | 4 | 144036127 | t | c | 0.5608 | -4.642 | 3.45E-06 | 0.5547 | -1.71 | 0.08726 | 0.5577 | -4.46 | 8.18E-06 |
| rs6808694 | 3 | 20186297 | t | c | 0.7309 | 4.42 | 9.89E-06 | 0.728 | 1.923 | 0.05451 | 0.7294 | 4.458 | 8.27E-06 |
| rs6805673 | 3 | 20185804 | a | g | 0.7271 | 4.374 | 1.22E-05 | 0.7238 | 1.944 | 0.05188 | 0.7254 | 4.441 | 8.94E-06 |
| rs9999826 | 4 | 144036457 | a | g | 0.4391 | 4.635 | 3.56E-06 | 0.445 | 1.678 | 0.09335 | 0.4421 | 4.433 | 9.30E-06 |
| rs11759394 | 6 | 47115212 | t | c | 0.9747 | -4.637 | 3.53E-06 | 0.9774 | -1.66 | 0.09688 | 0.9761 | -4.421 | 9.81E-06 |
| rs77215829 | 12 | 112618346 | a | c | 0.8808 | 4.519 | 6.21E-06 | 0.8751 | 1.689 | 0.09122 | 0.8775 | 4.225 | 2.39E-05 |
| rs138239252 | 3 | 41764014 | t | c | 0.1585 | -4.2 | 2.68E-05 | 0.172 | -1.71 | 0.08727 | 0.1658 | -4.105 | 4.05E-05 |
| rs56729913 | 3 | 20184853 | a | t | 0.2693 | -4.29 | 1.79E-05 | 0.2669 | -1.509 | 0.1312 | 0.2681 | -4.071 | 4.68E-05 |
| rs6807015 | 3 | 41754514 | t | c | 0.8337 | 4.074 | 4.62E-05 | 0.8291 | 1.769 | 0.07698 | 0.8312 | 4.063 | 4.85E-05 |
| rs10010247 | 4 | 138466213 | t | c | 0.5888 | -5.39 | 7.04E-08 | 0.5844 | -0.297 | 0.7664 | 0.5865 | -3.968 | 7.26E-05 |
| rs2711814 | 11 | 29376425 | t | c | 0.3867 | -4.608 | 4.07E-06 | 0.3874 | -1.054 | 0.2918 | 0.3871 | -3.966 | 7.31E-05 |
| rs2711824 | 11 | 29384629 | t | c | 0.6127 | 4.585 | 4.54E-06 | 0.6123 | 1.055 | 0.2914 | 0.6125 | 3.951 | 7.79E-05 |
| rs28695597 | 4 | 138458960 | a | c | 0.4098 | 5.233 | 1.66E-07 | 0.4151 | 0.401 | 0.6888 | 0.4125 | 3.933 | 8.40E-05 |
| rs12002585 | 9 | 81958890 | t | c | 0.928 | 4.146 | 3.39E-05 | 0.9264 | 1.353 | 0.176 | 0.9272 | 3.858 | 0.0001141 |
| rs56291600 | 17 | 38638260 | t | c | 0.9326 | -4.613 | 3.97E-06 | 0.9278 | -0.883 | 0.3772 | 0.9301 | -3.847 | 0.0001197 |
| rs2151155 | 11 | 29452159 | t | c | 0.3883 | -4.645 | 3.41E-06 | 0.3339 | -0.85 | 0.3956 | 0.3603 | -3.845 | 0.0001207 |
| rs10867369 | 9 | 81980758 | t | c | 0.0731 | -4.159 | 3.20E-05 | 0.0746 | -1.273 | 0.2031 | 0.0739 | -3.81 | 0.0001389 |
| rs17472276 | 17 | 38634605 | t | c | 0.9331 | -4.658 | 3.20E-06 | 0.9275 | -0.726 | 0.4679 | 0.9302 | -3.765 | 0.0001664 |
| rs72819648 | 17 | 38635876 | t | c | 0.067 | 4.662 | 3.13E-06 | 0.0725 | 0.721 | 0.4708 | 0.0698 | 3.765 | 0.0001667 |
| rs12551670 | 9 | 81957986 | a | c | 0.0725 | -4.035 | 5.46E-05 | 0.0736 | -1.287 | 0.1981 | 0.0731 | -3.734 | 0.0001885 |
| rs1575506 | 9 | 85788822 | a | g | 0.3229 | 4.512 | 6.44E-06 | 0.325 | 0.815 | 0.4148 | 0.324 | 3.728 | 0.0001933 |
| rs9817510 | 3 | 41813108 | t | c | 0.1676 | -3.941 | 8.10E-05 | 0.1556 | -1.417 | 0.1565 | 0.1611 | -3.714 | 0.0002039 |
| rs61493282 | 11 | 102754868 | t | c | 0.0788 | 4.41 | 1.04E-05 | 0.0818 | 0.859 | 0.3903 | 0.0803 | 3.688 | 0.0002261 |
| rs6967828 | 7 | 123254157 | t | c | 0.9192 | -4.778 | 1.77E-06 | 0.9218 | -0.414 | 0.6788 | 0.9205 | -3.625 | 0.000289 |
| rs76386877 | 4 | 138422617 | t | c | 0.613 | -5.539 | 3.03E-08 | 0.5862 | 0.336 | 0.7367 | 0.5992 | -3.617 | 0.0002978 |
| rs73718437 | 7 | 123253277 | t | c | 0.0809 | 4.808 | 1.52E-06 | 0.0779 | 0.352 | 0.725 | 0.0794 | 3.602 | 0.0003163 |
| rs6970517 | 7 | 123252866 | a | g | 0.0808 | 4.802 | 1.57E-06 | 0.0778 | 0.349 | 0.7271 | 0.0793 | 3.595 | 0.0003244 |
| rs13276017 | 8 | 134741381 | a | c | 0.2573 | 5.16 | 2.48E-07 | 0.2528 | -0.022 | 0.9828 | 0.255 | 3.578 | 0.0003458 |
| rs7865702 | 9 | 85790620 | t | c | 0.6731 | -4.387 | 1.15E-05 | 0.6702 | -0.715 | 0.4745 | 0.6716 | -3.569 | 0.0003585 |
| rs72983521 | 11 | 102730033 | t | c | 0.9216 | -4.265 | 2.00E-05 | 0.9205 | -0.744 | 0.4566 | 0.921 | -3.505 | 0.0004565 |
| rs113242701 | 8 | 73598626 | a | g | 0.9813 | -4.817 | 1.46E-06 | 0.9838 | -0.082 | 0.9346 | 0.9826 | -3.397 | 0.0006815 |
| rs60549974 | 12 | 112591821 | t | c | 0.9029 | 4.097 | 4.19E-05 | 0.9062 | 0.75 | 0.4531 | 0.9046 | 3.392 | 0.0006941 |
| rs186861709 | 8 | 73630793 | a | g | 0.0171 | 4.668 | 3.05E-06 | 0.0165 | 0.201 | 0.8403 | 0.0168 | 3.379 | 0.0007265 |
| rs2958828 | 8 | 134776896 | t | c | 0.2783 | 4.493 | 7.03E-06 | 0.275 | 0.335 | 0.7374 | 0.2766 | 3.37 | 0.0007517 |
| rs10107182 | 8 | 59392737 | t | c | 0.6623 | -4.13 | 3.63E-05 | 0.6526 | -0.685 | 0.4933 | 0.6573 | -3.368 | 0.0007563 |
| rs72985562 | 11 | 102800278 | t | g | 0.9195 | -4.152 | 3.30E-05 | 0.9172 | -0.61 | 0.5418 | 0.9183 | -3.33 | 0.0008697 |
| rs4738684 | 8 | 59393273 | a | g | 0.3401 | 4.045 | 5.24E-05 | 0.348 | 0.713 | 0.476 | 0.3442 | 3.329 | 0.0008726 |
| rs113145661 | 8 | 73510416 | c | g | 0.9794 | -4.634 | 3.59E-06 | 0.9829 | -0.231 | 0.8172 | 0.9813 | -3.327 | 0.0008782 |
| rs9297994 | 8 | 59392324 | a | g | 0.6614 | -4.09 | 4.32E-05 | 0.6522 | -0.633 | 0.5268 | 0.6567 | -3.303 | 0.0009578 |
| rs41276920 | 15 | 90347920 | a | g | 0.0798 | -3.96 | 7.51E-05 | 0.0764 | -0.755 | 0.45 | 0.078 | -3.3 | 0.000967 |
| rs2833496 | 21 | 33140026 | t | c | 0.2049 | 4.673 | 2.97E-06 | 0.2137 | 0.049 | 0.9611 | 0.2094 | 3.29 | 0.001003 |
| rs72754570 | 15 | 90348979 | a | g | 0.0834 | -4.176 | 2.96E-05 | 0.0801 | -0.5 | 0.6169 | 0.0817 | -3.284 | 0.001025 |
| rs204746 | 21 | 33139745 | c | g | 0.7951 | -4.676 | 2.93E-06 | 0.7862 | -0.032 | 0.9743 | 0.7905 | -3.28 | 0.001038 |
| rs9978172 | 21 | 33140566 | t | c | 0.2049 | 4.678 | 2.90E-06 | 0.2138 | 0.031 | 0.9756 | 0.2095 | 3.28 | 0.001038 |
| rs2922495 | 8 | 134735858 | t | c | 0.3205 | 4.607 | 4.08E-06 | 0.3218 | 0.022 | 0.9822 | 0.3212 | 3.225 | 0.00126 |
| rs7922120 | 10 | 115051007 | t | c | 0.0366 | 4.075 | 4.61E-05 | 0.0358 | 0.55 | 0.5822 | 0.0362 | 3.213 | 0.001315 |
| rs118187259 | 10 | 115121967 | t | c | 0.0121 | 4.009 | 6.10E-05 | 0.0103 | 0.675 | 0.4994 | 0.0111 | 3.185 | 0.001445 |
| rs7168849 | 15 | 90346227 | a | g | 0.9041 | 3.977 | 6.98E-05 | 0.9141 | 0.581 | 0.5609 | 0.9096 | 3.102 | 0.001922 |
| rs12218673 | 10 | 115053330 | t | c | 0.0378 | 4.026 | 5.67E-05 | 0.0351 | 0.425 | 0.6711 | 0.0364 | 3.088 | 0.002014 |
| rs8113355 | 19 | 49062163 | a | g | 0.7063 | -4.427 | 9.54E-06 | 0.7067 | 0.292 | 0.77 | 0.7065 | -2.835 | 0.00458 |
| rs8100256 | 19 | 49062157 | t | g | 0.7063 | -4.426 | 9.62E-06 | 0.7067 | 0.292 | 0.7703 | 0.7065 | -2.834 | 0.004593 |
| rs4981631 | 14 | 27312452 | a | c | 0.7508 | 4.65 | 3.33E-06 | 0.7514 | -0.581 | 0.5611 | 0.7511 | 2.822 | 0.004779 |
| rs1956818 | 14 | 27311163 | c | g | 0.262 | -4.584 | 4.57E-06 | 0.259 | 0.55 | 0.5825 | 0.2605 | -2.798 | 0.00514 |
| rs111955440 | 5 | 121004011 | t | c | 0.073 | -4.808 | 1.53E-06 | 0.0736 | 0.451 | 0.652 | 0.0733 | -2.789 | 0.005284 |
| rs3848542 | 19 | 49061724 | a | g | 0.2956 | 4.365 | 1.27E-05 | 0.2954 | -0.327 | 0.7435 | 0.2955 | 2.767 | 0.005655 |
| rs11696019 | 2 | 220664236 | t | c | 0.2147 | 4.393 | 1.12E-05 | 0.2113 | -0.46 | 0.6457 | 0.2129 | 2.73 | 0.006336 |
| rs982458 | 2 | 220663477 | t | c | 0.2138 | 4.35 | 1.36E-05 | 0.2118 | -0.468 | 0.6401 | 0.2128 | 2.694 | 0.007057 |
| rs13340342 | 5 | 120989002 | a | t | 0.9285 | 4.576 | 4.75E-06 | 0.9256 | -0.548 | 0.5836 | 0.9268 | 2.564 | 0.01034 |
| rs13340387 | 5 | 120986986 | t | c | 0.9286 | 4.42 | 9.85E-06 | 0.9257 | -0.484 | 0.6281 | 0.9269 | 2.511 | 0.01202 |
| rs6720347 | 2 | 220645599 | t | c | 0.7667 | -4.375 | 1.21E-05 | 0.7671 | 0.779 | 0.436 | 0.7669 | -2.489 | 0.01282 |
| rs111810710 | 15 | 24066424 | a | g | 0.0431 | 4.393 | 1.12E-05 | 0.0449 | -0.976 | 0.3291 | 0.0441 | 2.262 | 0.02372 |
| rs78186832 | 15 | 24069618 | t | c | 0.0436 | 4.403 | 1.07E-05 | 0.045 | -1.033 | 0.3017 | 0.0444 | 2.227 | 0.02597 |
| rs80245324 | 15 | 24069688 | a | t | 0.9564 | -4.404 | 1.06E-05 | 0.955 | 1.037 | 0.2997 | 0.9556 | -2.224 | 0.02614 |
| rs58673065 | 7 | 1885600 | a | g | 0.7641 | 4.022 | 5.76E-05 | 0.7464 | -0.823 | 0.4108 | 0.7544 | 2.098 | 0.03586 |
| rs11762803 | 7 | 1886805 | a | g | 0.7644 | 3.975 | 7.05E-05 | 0.7464 | -0.811 | 0.4171 | 0.7546 | 2.075 | 0.03803 |
| rs11766945 | 7 | 1888054 | a | g | 0.2137 | -4.385 | 1.16E-05 | 0.2264 | 1.445 | 0.1484 | 0.2206 | -1.882 | 0.05989 |
| rs2064738 | 14 | 27465839 | a | g | 0.7711 | 4.503 | 6.69E-06 | 0.7731 | -1.751 | 0.07992 | 0.7721 | 1.88 | 0.06009 |

CHR, chromosome; BP, base position; Freq1, frequency of Allele1
